# Biocatalytic Potential of a Mycobacterial Aminoacylase for Synthesis of *N‐*Acyl‐L‐Amino Acids in Aqueous Media

**DOI:** 10.1002/cbic.70313

**Published:** 2026-04-08

**Authors:** Jessika Wirges, Gerrit Haeger, Laureline Gennesseaux, Yann Guiavarc’h, Catherine Humeau, Cédric Paris, Isabelle Chevalot, Karl‐Erich Jaeger, Jonas Krapohl, Patrick Schmidt, Johannes Bongaerts, Petra Siegert

**Affiliations:** ^1^ Institute of Nano‐ and Biotechnologies Aachen University of Applied Sciences Jülich Germany; ^2^ Novonesis A/S, Production Strain Technology Bagsværd Denmark; ^3^ Laboratoire Réactions et Génie des Procédés (LRGP) CNRS Université de Lorraine Nancy France; ^4^ Laboratoire d’Ingénierie des Biomolécules (LIBio) CNRS Université de Lorraine Nancy France; ^5^ Institute of Molecular Enzyme Technology Heinrich Heine University Düsseldorf Jülich Germany

**Keywords:** acylation, amino acids, aminoacylase, biocatalysis, biosurfactants, molecular docking

## Abstract

In this study, we present an investigation of the recently identified aminoacylase MsAA for the synthesis of *N*‐acyl‐L‐amino acids, focusing on *N*‐lauroyl‐L‐methionine. We found optimal reaction conditions at pH 8.0 and a temperature of 40–45°C with substrate concentrations of 400 mM methionine and 150 mM lauric acid. The highest product concentration of 100 mM was achieved with 67% substrate conversion after 72 h reaction at 40°C and pH 8.0. The reaction could be upscaled with a nearly identical reaction course. Several other fatty acids were also found to be substrates of this enzyme. Besides methionine, only hydrophobic amino acids were accepted for acylation. For a detailed analysis of the catalytic reaction mechanism of MsAA, we performed in silico protein modeling studies. Molecular docking of lauric acid and methionine to the predicted MsAA structure resulted in a similar mode of substrate binding as described for the related *N*‐succinyl‐L,L‐diaminopimelic acid desuccinylase from *Haemophilus influenzae*. Differences in amino acid sequence of structurally conserved substrate‐binding residues explain the distinct substrate scope of the enzymes. The structure–function relationship of relevant amino acid residues was validated by a mutagenesis study.

## Graphical abstract

The aminoacylase MsAA is a suitable enzyme for the biotechnological production of *N*‐acyl‐L‐amino acids. This study focuses on the optimization of the synthesis of *N*‐lauroyl‐L‐methionine. A detailed analysis of the catalytic reaction mechanism of MsAA is performed in an in silico protein modeling study.

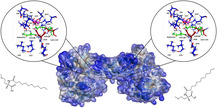

## 1 Introduction

Acyl amino acids are valuable compounds for cosmetic products. They consist of an amino acid linked to a fatty acid, most commonly through *N‐*acylation under the formation of an amide bond, but also through *O*‐acylation by ester formation. Most commonly lauric acid is used, or fatty acids derived from coconut oil, which is rich in lauric acid. Amino acid‐based surfactants are considered as remarkably skin‐friendly due to their low inflammatory potential. In high‐grade cosmetic products, *N*‐lauroyl‐L‐glutamic acid is commonly used as a surfactant [1].

Even though the products are of plant or other biological origin, and can thus be classified as biosurfactants, chemical synthesis of these compounds is typically achieved by the environmentally harmful Schotten–Baumann synthesis. For this acylation method, acyl chlorides are necessary, rendering the synthesis not compliant with green chemistry principles [2]. First, fatty acid chlorides must be synthesized, which can be done using phosgene, thionyl chloride, or phosphoryl trichloride. All of these are especially harmful chemicals. The fatty acyl chlorides are particularly reactive and can hydrolyze, resulting in the formation of hydrogen chloride, which poses an additional hazard. Furthermore, during the acylation reaction, sodium chloride is stoichiometrically produced as waste. Substituting harmful acyl chlorides with coupling agents used in peptide synthesis is possible and performed at laboratory‐scale synthesis, but this approach is often not feasible at industrial scale due to cost constraints [3, 4].

Biocatalytic synthesis using enzymes is therefore a promising alternative because it can be performed with unmodified, free fatty acids, as recently reviewed [4]. This eliminates the need for chlorination and prevents the formation of waste products. Aminoacylases have been described and investigated for this purpose but are widely unexplored and underexploited for commercial applications. They catalyze the reversible hydrolysis of *N*‐acylated amino acids, mainly proteinogenic L‐amino acids at the α‐position. However, ϵ‐lysine aminoacylases have been described as well [5]. Aminoacylases have been identified from various organisms [6, 7, 8, 9], especially in *Streptomycetes*. A variety of aminoacylases have been shown to be applicable for biocatalytic synthesis [10, 11, 12, 13, 14, 15]. The broad‐spectrum α‐aminoacylase SamAA from crude extract of *Streptomycetes ambofaciens* has been used for acylation of a variety of amino acids, with conversion rates reaching 30 % for α‐lauroyl‐lysine [14]. The acylation reaction catalyzed by SamAA was optimized for 10‐undecenoyl‐phenylalanine, revealing a strong dependence of pH and substrate concentration. Optimal activity was observed at pH 8.0 and an equimolar substrate concentration of 100 mM. Also, the recently described homologs aminoacylase SgAA from *Streptomycetes* *griseus* yielded biocatalytic synthesis of *N*‐lauroyl‐L‐methionine. However, under non‐optimized conditions, the reaction yielded a conversion of only 4% lauroyl‐L‐methionine [16]. Higher conversions were achieved with the porcine aminoacylase‐1 (pAcy1), which was tested in a glycerol–water system. The enzyme yielded lauroyl‐L‐arginine and lauroyl‐L‐glutamic acid at 82% and 44% conversion respectively [17]. Another exceptional aminoacylase is the long‐chain‐acyl aminoacylase from *Paraburkholderia monticola* (PmAcy). It catalyzed the acylation of several amino acids, the highest conversion was achieved in the synthesis of lauroyl‐L‐histidine (62%) and lauroyl‐L‐arginine (61%) [13]. However, there are a number of obstacles to an economical process, in particular the difficulty of recombinant expression of the enzymes and their low stability [15]. Recently, we identified the mycobacterial aminoacylase MsAA from *Mycolicibacterium smegmatis* MKD 8 and optimized its expression in *Escherichia* *coli* and *Vibrio natriegens* [18]. In a first screening for acylation of proteinogenic L‐amino acids, *N*‐lauroyl‐L‐methionine, *N*‐lauroyl‐L‐isoleucine, *N*‐lauroyl‐L‐leucine, *N*‐lauroyl‐L‐valine, *N*‐lauroyl‐L‐alanine, and *N*‐lauroyl‐L‐phenylalanine were synthesized with product concentrations of 7.4, 5.8, 5.1, 4.5, 1.1, and 0.1 mM, respectively [18].

MsAA belongs to the M20A family of metallopeptidases, which are characterized by a cocatalytic active site, most often with two zinc ions, ligated by histidine and glutamic acid residues, with an aspartic acid residue bridging the zinc ions [19]. Some enzymes from the M20A family of metallopeptidases have been thoroughly characterized and their crystal structures have been published. A homodimeric structure has been found for *N*‐succinyl‐L,L‐diaminopimelic acid desuccinylase HiDapE from *Haemophilus influenzae* [20] (P44514), which is a homologous enzyme to MsAA and for carboxypeptidase G2 from *Pseudomonas* sp [21]. The structural studies enabled not only the proposal of a catalytic mechanism for hydrolysis, but also revealed dynamic movement of the dimeric units during substrate binding and release. The crystal structure of HiDapE with bound substrates has been solved [22]. The tertiary structure can be divided into a catalytic domain and a dimerization domain linked by a flexible hinge, which closes and opens with substrate binding and release. Closing the structure brings the monomers closer together at each catalytic site, playing a role in catalytic mechanism and substrate binding. During hydrolysis, the nonmetal binding, catalytic glutamate E134(A) residue acts as a general base and can deprotonate a zinc‐bound water molecule. The formed hydroxide ion can attack the substrate's carbonyl carbon atom, leading to a tetrahedral intermediate. This negatively charged transition state is stabilized by histidine H194(B) from the opposing dimer, forming an oxyanion hole [22]. This suggests that the monomeric protein is not catalytically active. Upon decomposition to the hydrolyzed products, the substrate is released.

During the initial screening of MsAA for acylation activity, *N*‐lauroyl‐L‐methionine was preferentially synthesized. A conversion of 7.4% after 72 h from 100 mM lauric acid and 100 mM methionine in 100 mM Tris‐HCl at pH 7.0 and 40°C was initially achieved [18]. Here, we present a significant optimization of the acylation conditions resulting in a final concentration of 100 mM *N*‐lauroyl‐L‐methionine and 67% substrate conversion. The optimization of several reaction parameters resulted in a 13.5‐fold increase of product concentration and a 9.0‐fold increase in conversion rate of the substrate lauric acid.

Motivated by these results, we aimed to gain further insight into the biocatalytic acylation of methionine on a molecular level. Analysis of the protein structure of MsAA predicted by AlphaFold [18, 23], and docking experiments were conducted. Comparison to HiDapE revealed several similarities, such as domain architecture and relative positioning of the active site. Therefore, we performed molecular docking of lauric acid and methionine. The docking results show major differences in substrate‐binding residues that explain the distinctive substrate scope of these enzymes.

## 2 Results and Discussion

### 2.1 Enzyme Production, Hydrolytic, and Synthetic Activity

The aminoacylase MsAA was recombinantly produced in *E. coli* and purified to homogeneity as described previously [18]. Hydrolytic activity against 15 mM acetyl‐alanine was 130 U/mg (standard activity assay, in 100 mM Tris‐HCl pH 7.0, 30°C). As described in a previous study, hydrolytic pH optimum was at pH 7.0 and the enzyme was stable up to 40°C. These conditions were chosen to initially investigate the catalytic potential of acylation of L‐amino acids. As a screening to probe the acceptance of proteinogenic amino acids, equimolar substrate concentrations of 100 mM lauric acid and amino acid were used. Only small, hydrophobic amino acids were accepted. *N*‐lauroyl‐L‐methionine was produced with the highest yield [18]. Therefore, we choose this amino acid for optimization of the acylation reaction.

### 2.2 Determination of pH and Temperature Optimum

**FIGURE 1:**
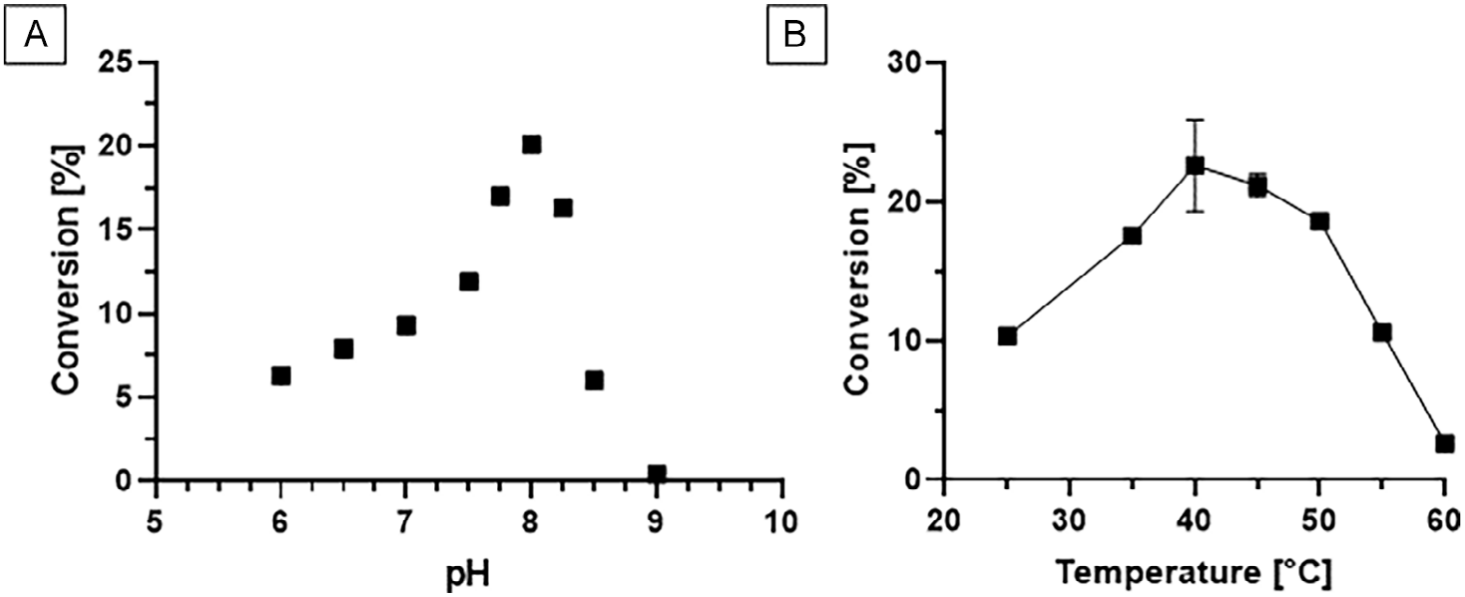
pH and temperature dependency of biocatalytic *N*‐lauroyl‐L‐methionine synthesis with the aminoacylase MsAA. (A) pH dependency of *N*‐lauroyl‐L‐methionine synthesis. The reactions were conducted at 40°C for 72 h with 100 mM methionine and 100 mM lauric acid in 100 mM Tris‐HCl at various pH. All reactions were conducted in triplicates. (B) Temperature dependency of *N*‐lauroyl‐L‐methionine synthesis. The reactions were conducted for 72 h with 100 mM methionine and 100 mM lauric acid in 100 mM Tris‐HCl at pH 8.0 at various temperatures. All reactions were conducted in triplicates. Error bars under 0.7% are not displayed.

The influence of pH on the conversion rate was investigated to find optimal conditions for the synthesis of *N*‐lauroyl‐L‐methionine. In contrast to the hydrolytic pH profile, optimal synthesis reaction was observed at pH 8.0 with 20.1% conversion (Figure 1A). The synthesis is highly sensitive to changes of pH. At pH 7.0, only half of the conversion was detected, and at pH 9.0, no synthetic activity was detected at all. The latter can be explained by insufficient stability of the enzyme at pH 9.0 [18]. Hence, further optimization of the synthesis reaction was conducted at pH 8.0. The slight change of pH optimum to more basic pH values from hydrolysis to synthesis may be explained by higher nucleophilicity of the amino group of methionine at higher pH values. An optimal pH of 8.0 for the acylation of phenylalanine was also determined for the homologous aminoacylase SamAA from *S. ambofaciens* [15]. The optimal temperature for hydrolysis was measured to be 70°C [18]. However, as the synthesis reaction is much slower, insufficient stability can counteract activity. Thus, the temperature optimum was again determined for synthesis in a range of 25°C to 60°C and conversion was measured after 72 h. After 24 h, the optimal reaction temperature was 45°C with a conversion of 21.3% and did not further increase after 72 h (data not shown). At 40°C, conversion after 24 h was 14.7% and 22.6% after 72 h (Figure 1B). Conversion rates at 40°C and 45°C are roughly in the same range after 72 h. The optimal temperature for synthesis with SamAA was 45°C, and the enzyme quickly lost its activity at 55°C [15].

### 2.3 Determination of Optimal Substrate Concentrations

**FIGURE 2:**
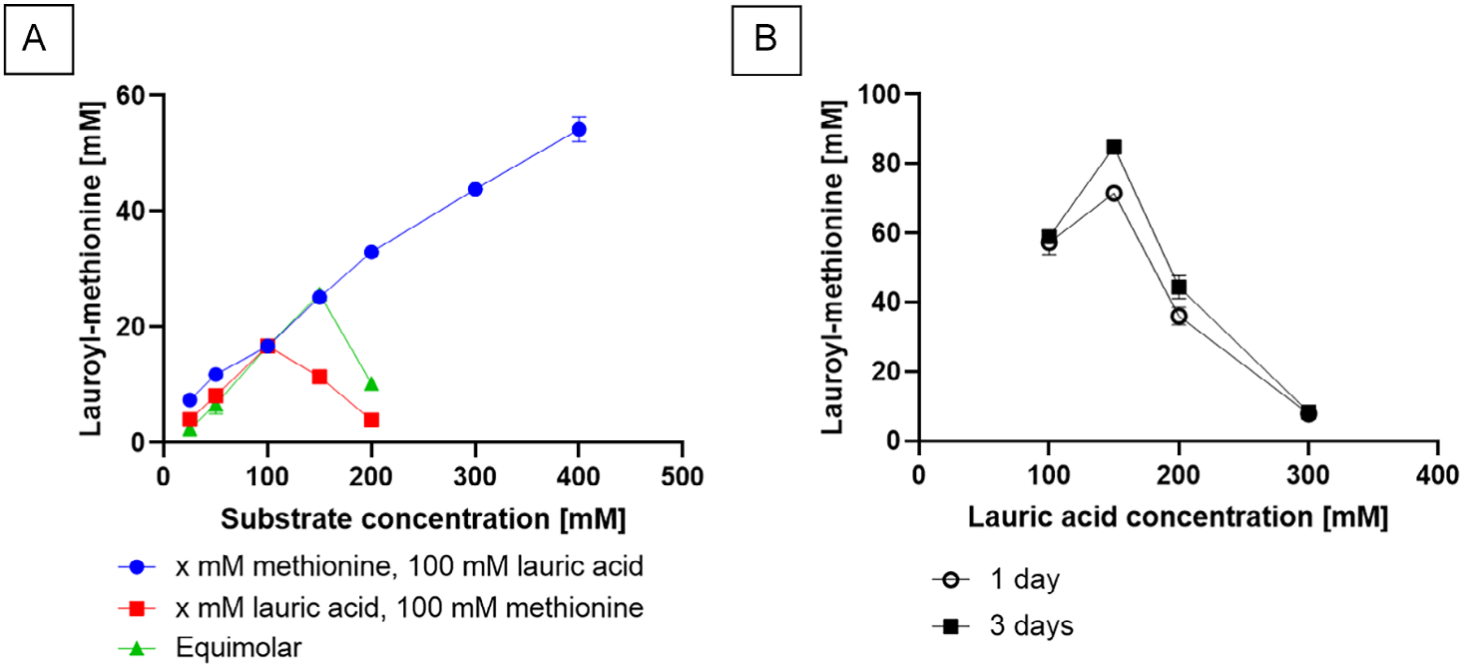
*N*‐lauroyl‐L‐methionine synthesis using the aminoacylase MsAA at varying substrate conditions. (A) Synthesis of *N*‐lauroyl‐L‐methionine with varying concentration of methionine and lauric acid. Product formation was tested with 100 mM lauric acid and 25–400 mM methionine (blue), 100 mM methionine and 25–200 mM lauric acid (red), and at equimolar conditions from 25–200 mM (green). Reactions were conducted at pH 8.0 and 45°C for 24 h. (B) Synthesis with 400 mM methionine and 100–300 mM lauric acid at pH 8.0 and 40°C after 24 and 72 h. All reactions were conducted in triplicates. Error bars under 2 mM are not displayed.

After determination of the pH and temperature profiles of MsAA with both substrates used equimolarly at 100 mM, the optimal substrate concentrations for the synthesis were determined. In general, one of the substrates is present in excess toward the other in order to perform acylation by aminoacylases. The concentration of one substrate was kept constant at 100 mM, while the other substrate was set to 25, 50, 100, 150, or 200 mM. Furthermore, the reaction of 300 and 400 mM methionine was tested with lauric acid at 100 mM. Reactions were conducted at equimolar ratios at the same concentration increments as well. At 400 mM methionine, various concentrations of lauric acid were investigated. An increasing methionine concentration had the strongest positive effect on product formation. With 100 mM lauric acid, increasing the methionine concentration steadily also increased the formation of *N*‐lauroyl‐L‐methionine. An excess of lauric acid inhibited the reaction: The methionine concentration should thus be adjusted to the lauric acid concentration (Figure 2A). The highest concentrations of *N*‐lauroyl‐L‐methionine were determined at 400 mM methionine and 150 mM lauric acid with 84.9 mM product and 56.6% conversion (Figure 2B).

### 2.4 Upscaling of the Enzymatic Acylation

**FIGURE 3:**
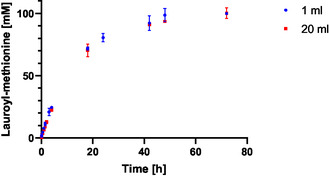
Time course of the biocatalytic synthesis reaction of *N*‐lauroyl‐L‐methionine in 1 and 20 ml scale. The reaction was conducted with 400 mM methionine and 150 mM lauric acid in 100 mM Tris‐HCl pH 8.0 at 40°C in either 1.5 mL reaction tubes with 1 mL filling volume or in Wheaton Cellstir reactors with 20 mL filling volumes. The tubes were shaken on an orbital shaker with 500 rpm, and the Cellstir reactors were stirred with 500 rpm. Error bars under 1 mM are not displayed.

In order to test if the biocatalytic conversion and the reaction course remained consistent between different reactor types, an upscaling to a stirred enzyme reactor was performed. The small‐scale reaction was conducted with 1 mL total reaction volume in 1.5 mL reaction tubes at 500 rpm. The larger scale consisted of 20 mL reaction volume in a 100 mL stirred reactor. The reaction was conducted at 40°C with 400 mM methionine and 150 mM lauric acid. In both scales, product formation over the reaction course was almost identical, indicating that results obtained from small‐scale reaction tube screening can be transferred to the dimensions of a lab‐scale stirred tank reactor. Maximal product formation was observed after 72 h with 100.2 mM *N*‐lauroyl‐L‐methionine, which is a conversion rate of 67% relative to lauric acid (Figure 3). Compared to the initial product concentration and conversion rate of 7.4 mM and 7.4% [18], the optimization of reaction parameters resulted in a 13.5‐fold increase of product concentration and a 9.0‐fold increase in conversion rate. To the best of our knowledge, this represents the highest conversion rate to *N*‐lauroyl‐L‐methionine for aminoacylases to date [4]. At the optimized conditions, the addition of glycerol demonstrated a stabilizing effect on the enzyme; however, overall conversions were lower than in the absence of glycerol (Table S1).

### 2.5 Optimization of Acylation With Different Proteinogenic Amino Acids

**FIGURE 4:**
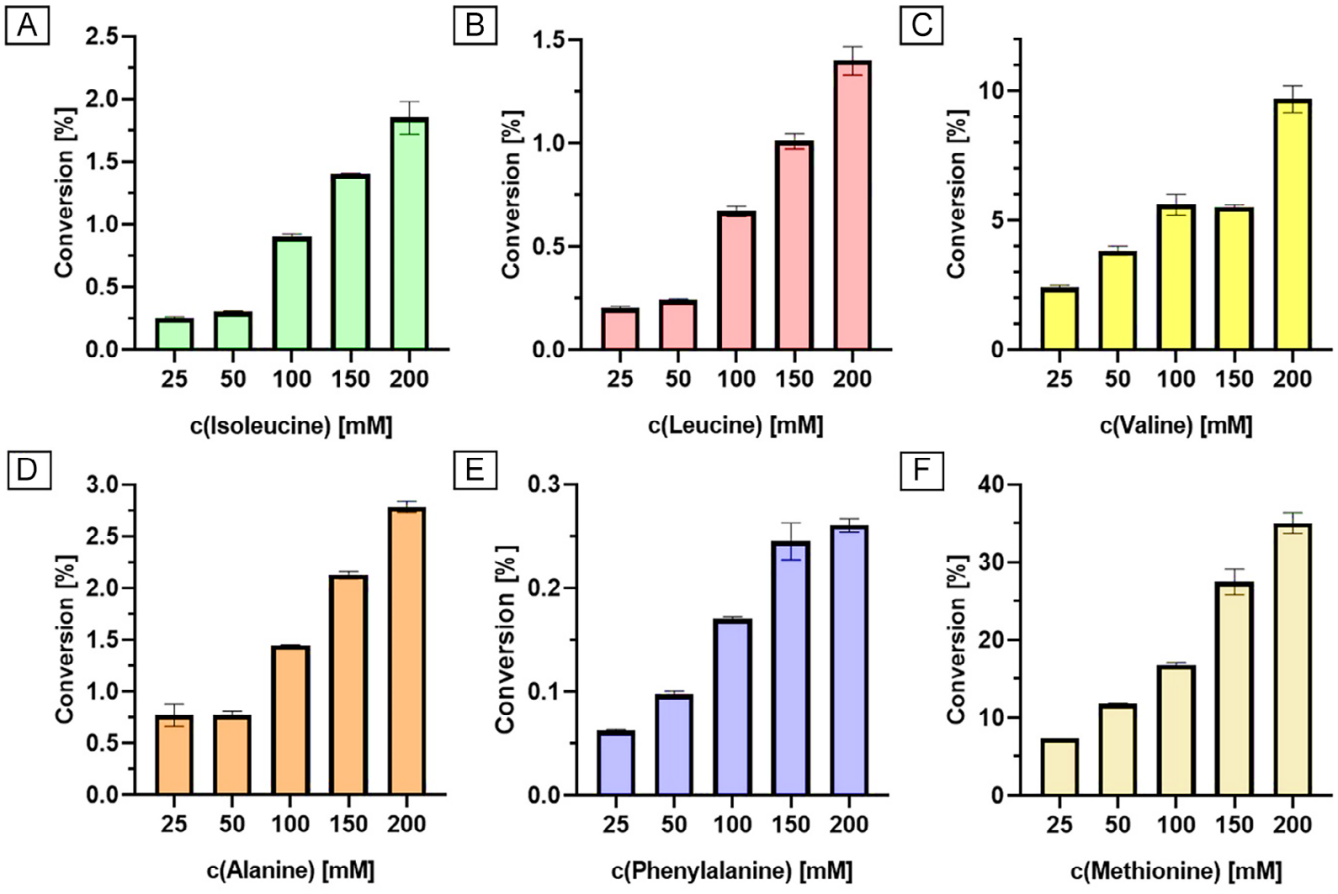
*N*‐lauroyl‐L‐amino acid synthesis with the aminoacylase MsAA at varying substrate conditions. Synthesis of N‐lauroyl‐L‐isoleucine (A), and ‐leucine (B), ‐valine (C), ‐alanine (D), ‐phenylalanine (E), and ‐methionine (F) with varying concentration of the respective amino acid and 100 mM lauric acid at pH 8.0 and 45°C for 24 h. All reactions were conducted in triplicates. Error bars under 1% are not displayed.

After finding the optimized acylation conditions for methionine, other acyl acceptors were investigated. In contrast to the initial substrate scope, which was conducted at pH 7.0 [18], the pH of the reactions was set to pH 8.0. The reaction was conducted at 45°C for 24 h. No further acylated amino acid products were detected for acylation by altering the pH value to pH 8.0 (data not shown). It is worth to mention that he homologous aminoacylases from *S. ambofaciens* exhibited a broader substrate scope, with efficient acceptance of nonpolar as well as polar acyl acceptors. Especially lysine and arginine yielded the highest conversion with 10‐undecenoic acid and 24% conversion yield [14]. For MsAA, it was investigated if the conversion of the accepted amino acids, namely, alanine, isoleucine, leucine, phenylalanine, and valine could be increased by varying the amino acid concentration as well. The amino acids were used in concentrations from 25 to 200 mM, which was their limit of solubility in aqueous buffer. In all cases, the conversion rate could be increased by higher amino acid concentration up to 200 mM. The highest product concentrations for *N*‐lauroyl‐L‐alanine, ‐isoleucine, ‐leucine, ‐phenylalanine, and ‐valine were 2.8, 1.9, 1.4, 0.26, and 9.7 mM, respectively (Figure 4A–F). Hence, valine was the second‐best substrate for acylation, besides methionine, which yielded 35.0 mM product under the same condition. All lauroyl‐amino acids were detected by LC‐MS (Figure S1‐5; S9).

### 2.6 Acyl‐Donor Specificity for Acylation of Methionine

The acyl‐donor specificity for the acylation of methionine was investigated with caprylic acid, decanoic acid, undecanoic acid, 10‐undecenoic acid, myristic acid, palmitic acid, and oleic acid. The reactions were conducted at 40°C and pH 8.0 for 72 h, at an amino acid concentration of 200 mM and fatty acid concentration at 100 mM. To increase the substrate solubility, 10% EtOH (v/v) was added to the substrate solutions. The acylation with lauric acid served as a reference to estimate product concentrations for the remaining aliphatic fatty acids. With all investigated fatty acids, product could be detected by high performance liquid chromatography‐mass spectrometry (HPLC)‐(MS) (Figure S6‐S11), albeit cinnamic acid and ferulic acid were not accepted as substrates by MsAA. Compared *N*‐lauroyl‐L‐methionine, the acylation of methionine with caprylic acid, decanoic acid, undecanoic acid, 10undecenoic acid, myristic acid, palmitic acid, and oleic acid were 71.1%, 85.0%, 68.3%, 106.5%, 96.0%, 3.3%, and 2.0%, respectively (Table 1). The results are in line with the results reported for the homologous aminoacylases from *S. ambofaciens*, where lauric acid and undecenoic acid were the preferred acyl donors, while acylation with caprylic acid and oleic acid yielded lower relative conversions [14].

**TABLE 1:** Acyldonor specificity for the acylation of methionine. The reactions were conducted with 200 mM methionine and 100 mM fatty acid at pH 8.0 and 40°C for 72 h. Ethanol was added at 10% (v/v). 100% *N*‐lauroyl‐L‐methionine corresponds to 20.1 mM. An evaporative light scattering detector (ELSD) was used.

Fatty acid	Peak area (ELSD), %
Caprylic acid (C8:0)	71.1 (±6.1)
Decanoic acid (C10:0)	85.0 (±12.0)
Undecanoic acid (C11:0)	68.3 (±0.7)
10‐Undecenoic acid (C11:1)	106.5 (±2.5)
Lauric acid (C12:0)	100 (±1.2)
Myristic acid (C14:0)	96.0 (±2.6)
Palmitic acid (C16:0)	3.3 (±0.3)
Oleic acid (C18:1)	2.0 (±1.1)

### 2.7 Molecular Modeling of Substrate Binding to MsAA

The aminoacylase MsAA can be assigned to the M20A metallopeptidase family, characterized by its metal‐binding residues (H91, D123, E158, E185, and H425) for the cocatalytic zinc site, as well as conserved catalytic residues (D93, E157, and H226), as in HiDapE [20, 22] and the homologous aminoacylases from *S. ambofaciens* (SamAA) [18], *Streptomyces* *mobaraensis* (SmAA) [24] (C9K2Z6), *S. griseus* (SgAA) [16], and the pAcy1 [25] (Table 2). The residue D93 is considered as catalytic, because it can form interactions between the Nϵ‐proton of H91, assisting in orientation and influencing the electronegativity of the Nϵ‐nitrogen, leading to a decreased Lewis acidity of the zinc ion [20, 27]*.*

**TABLE 2:** Functional amino acids of MsAA and its homologs and their proposed functions.

MsAA	SmAA	SgAA	hAcy1	HiDapE	Function
H91	H107	H90	H80	H67	Metal‐binding [18, 20]
D123	D139	D122	D113	D100	
E158	E174	E157	E148	E135	
E185	E201	E184	E175	E163	
H425	H435	H418	H373	H349	
D93	D109	D92	D82	D69	Catalytic, orientation of H67_HiDapE_ [20], or H91_MsAA_ (not shown for hAcy1)
E157	E173	E156	E147	E134	Catalytic, acid–base catalyst [22]
H226	H238	H221	H206	H194	Catalytic, oxyanion hole‐forming [22, 26]; not essential in pAcy1 [25]
L213	M225	M208	P193	S181	Amino acid binding in HiDapE [22]
L357	D367	D350	N307	S290	
W215	W227	W210	W195	T183	Amino acid binding
R328	R338	R321	R276	R258	Binding of α‐carboxylic acid [22, 26]
K397	K407	K390	R348	R329	Binding of α‐carboxylic acid in hAcy1 [25]; Possibly amino acid binding in HiDapE [20]
M229	M241	M224	R209	Y197	Binding of succinic acid (carboxyl) in HiDapE [22]; Acyl‐binding in MsAA; hAcy1 unknown
E210	Q222	Q210	E190	R178	Binding of succinic acid (carboxyl) in HiDapE
N315	N325	N308	N263	N245	Binding of amino acid at α‐carboxylic group in HiDapE [22], hAcy1 [26], and MsAA
A314	V324	V307	Y262	N244	Binding of amino acid at ϵ‐carboxylic group in HiDapE [22]
D326	D336	D319	D274	N256	Orientation of arginine residue (R276_hAcy1_) [26]

Furthermore, a dimeric protein structure has been described for MsAA [18], HiDapE [22], and pAcy1 [28]. In contrast, SmAA has been described as a monomeric enzyme [24] and the potential oligomeric state of SamAA has not yet been determined. The aminoacylase MsAA shows 21.4% sequence identity to HiDapE, 25.5% identity to pAcy1, 25.2% identity to the human aminoacylase‐1 (hAcy1) [26] (Q03154), 56.5% to SgAA, 56.3% to SamAA, and 54.9% identity to SmAA, respectively. Despite sharing similarities with the aminoacylases SmAA and pAcy1, HiDapE does not hydrolyze N‐acetyl‐amino acids but catalyzes the hydrolysis of N‐succinyl‐L,L‐diaminopimelic acid to succinate and L,L‐diaminopimelic acid [29]. HiDapE has been well studied with regard to structural and mechanistic features and its structure (PDB 5VO3) with the products succinic acid and diaminopimelic acid has been solved by X‐ray crystallography [22]. Due to the conserved catalytic and metal.‐binding residues between MsAA and HiDapE, it seemed worthwhile to compare these enzymes. The experimental investigation of the acylation of amino acids with MsAA revealed that the enzyme has a strong preference for methionine and accepts only hydrophobic amino acids as substrates for acylation. With the protein structure predicted by AlphaFold, we performed docking experiments to comprehend this behavior and understand the favored binding of hydrophobic amino acid substrates, especially methionine, in the acylation reaction.

**FIGURE 5:**
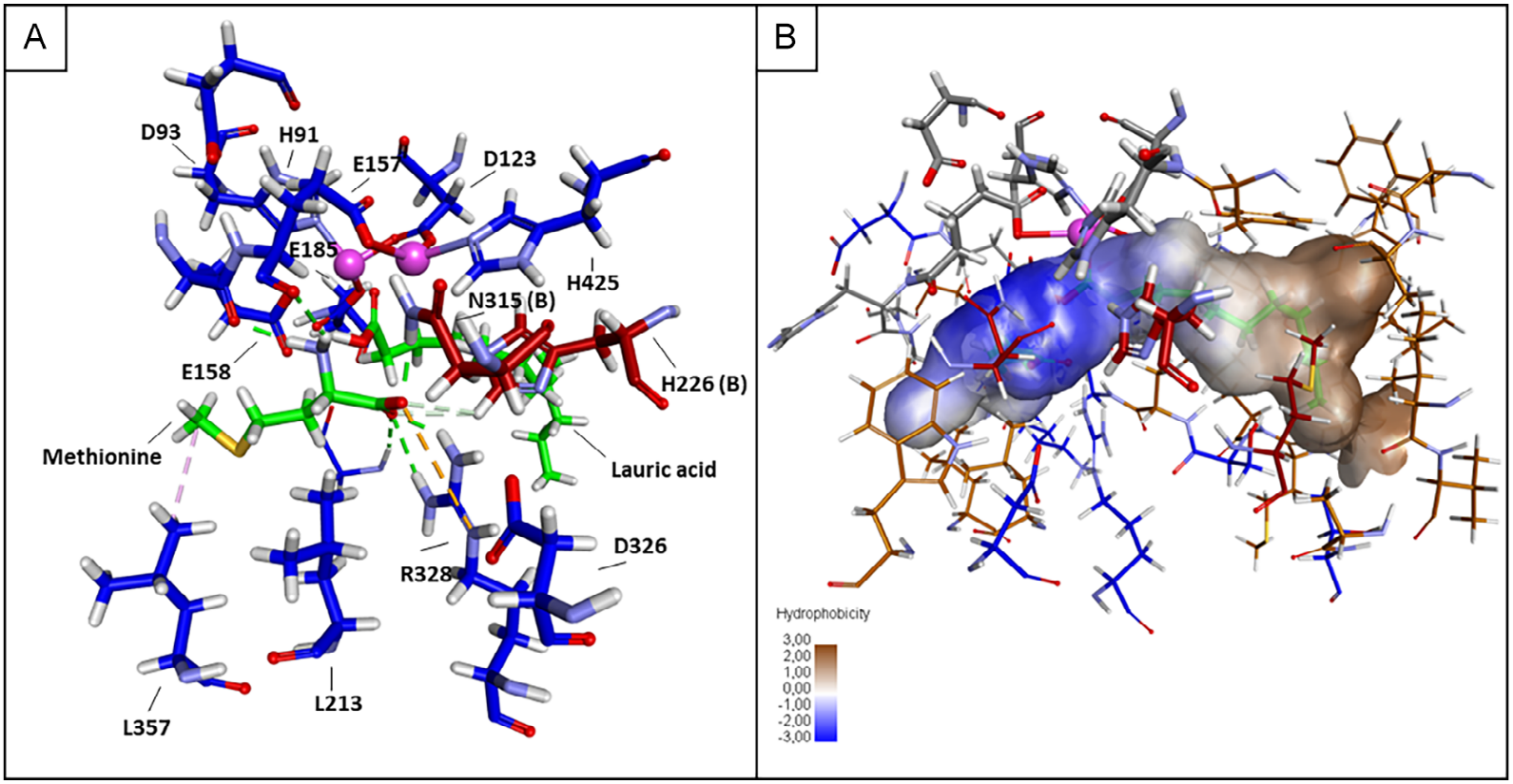
Results obtained from docking experiments with methionine and lauric acid to MsAA. (A) Docking of lauric acid and methionine to the active site of dimeric MsAA. Zinc ions are shown as magenta balls. The substrates methionine and lauric acid are shown with green carbon atoms. The dimers A and B are shown with blue and red carbon atoms, respectively. The ligand interactions are shown as dashed lines: green for H‐bonds, orange for electrostatic interactions and lilac for hydrophobic interactions. (B) Substrate binding site with highlighted hydrophilic and hydrophobic pockets. Hydrophobic regions and amino acids are shown in brown color, hydrophilic regions and residues are shown in blue. Residues of the active site are shown with gray carbon atoms, and residues from the adjacent second monomer are shown with red carbon atoms.

For MsAA, both substrates, lauric acid and methionine, could be docked to the active sites of the dimeric enzyme and catalytic as well as substrate binding residues were visualized (Figure 5A). All conserved residues from HiDapE, hAcy1, SmAA, SgAA, and MsAA are listed in Table 2 with their corresponding (postulated) function. The carbonyl oxygen of lauric acid is positioned between the two zinc ions. The methionine molecule is positioned with its amino group facing the carbonyl group of lauric acid. In the docking experiment, interactions were established between the catalytic residue E157_MsAA_ (Figure 5A) with both methionine and lauric acid and distances between these groups are <2.9 Å. Therefore, the glutamic acid residue might act in deprotonation of the amino group of methionine and protonation of the carboxyl group of lauric acid, as it is suggested for HiDapE. The interaction with E157_MsAA_ with both the amino group of methionine and the carboxylic group of lauric acid suggests the role as a general acid/base in catalysis [30].

Regarding substrate binding residues in MsAA, N315(B) _MsAA_ of the second monomer binds the amino acid at the α‐carboxylic group (Figure 5A), and this residue is conserved in HiDapE as N245(B)_HiDapE_ as well as in hAcy1 as N263(B)_hAcy1_ [26]. The residue is also conserved in SmAA as N325; however, this enzyme has been described to be monomeric [24]. The presence of α‐carboxyl groups in both acyl‐amino acids as well as in diaminopimelic acid could explain the similarities of substrate binding. In HiDapE, adjacent to N245(B)_HiDapE_, a second N244(B)_HiDapE_ binds the ϵcarboxylic group of diaminopimelic acid. This N244(B) is not conserved in MsAA but exchanged to a hydrophobic A314(B)_MsAA_. This might explain why MsAA does not accept e.g., glutamic acid as a substrate for acylation.

Furthermore, in HiDapE, Y197(B)_HiDapE_ of the second monomer, oriented in proximity to H194(B)_HiDapE_, participates in substrate binding, namely the non‐amide forming carboxylic group of the succinic acid moiety [22]. This tyrosine residue is exchanged to methionine (M229_MsAA_) in MsAA and participates in binding of lauric acid by hydrophobic interactions (Table 2). In HiDapE, the same carboxylic group of succinic acid also interacts with R178_HiDapE_ but is exchanged to E210_MsAA_ in MsAA. This glutamic acid residue is however not structurally conserved and does not participate in substrate binding in MsAA docking.

In HiDapE, R258_HiDapE_ binds the α‐carboxyl group of diaminopimelic acid, both this residue and its function are conserved in MsAA as R328_MsAA_ and in hAcy1 as R276_hAcy1_ (Figure 5A). Furthermore, for hAcy1 D274_hAcy1_ assists in orientation of R276_hAcy1_, possibly through a salt bridge, and a D274A_hAcy1_ variant exhibits strongly reduced activity [26]. This aspartic residue is conserved in MsAA as D326_MsAA_ and might similarly interact with R328_MsAA_ (Figure 5A). There is some discrepancy for the substrate binding role of R276_hAcy1_ in hAcy1, as it is shown to not be essential, and R348_hAcy1_ has been postulated for substrate binding [25]. This residue is not conserved in MsAA, where it is exchanged to K397_MsAA_. However, R276_hAcy1_ is conserved in HiDapE as R329_HiDapE_ and interacts with the ϵcarboxyl group of diaminopimelic acid. For hAcy1, a R348A_hAcy1_ variant was not hydrolytically active; hence, it was suspected to be involved in substrate binding[25]. Liu et al*.* published the docking of *N*‐acetyl‐L‐methionine to a homology model of hAcy1, which indicated hydrogen bonding of R348_hAcy1_ to the α‐carboxylic acid and amide‐carbonyl groups [25], suggesting a similar role of R258_HiDapE_ in HiDapE and R328_MsAA_ in MsAA.

In Figure 5B, the substrate pocket is visualized. A clear partitioning into a hydrophilic and a hydrophobic region for amino acid and fatty acid binding, respectively, can be observed. Furthermore, oriented below the side chain of methionine, two structurally adjacent leucine residues, L213_MsAA_ and L357_MsAA_, participate in substrate binding, which may be responsible for the specificity of MsAA for methionine and hydrophobic amino acids in general. These two leucine residues are not conserved in the homologous enzymes SamAA, SmAA, hAcy1, or HiDapE [22] which do not exhibit a bias toward hydrophobic amino acids. In HiDapE protein structure, in place of these two hydrophobic leucine residues, two serine residues can be found, which can interact with the amino‐ and carboxylic groups of diaminopimelic acid. We probed these positions by site‐directed‐mutagenesis and MsAA variants with changed substrate specificity are currently under investigation in our lab (data not shown).

### 2.8 Docking Analysis of the Tetrahedral Intermediate and Reaction Product in MsAA

**FIGURE 6:**
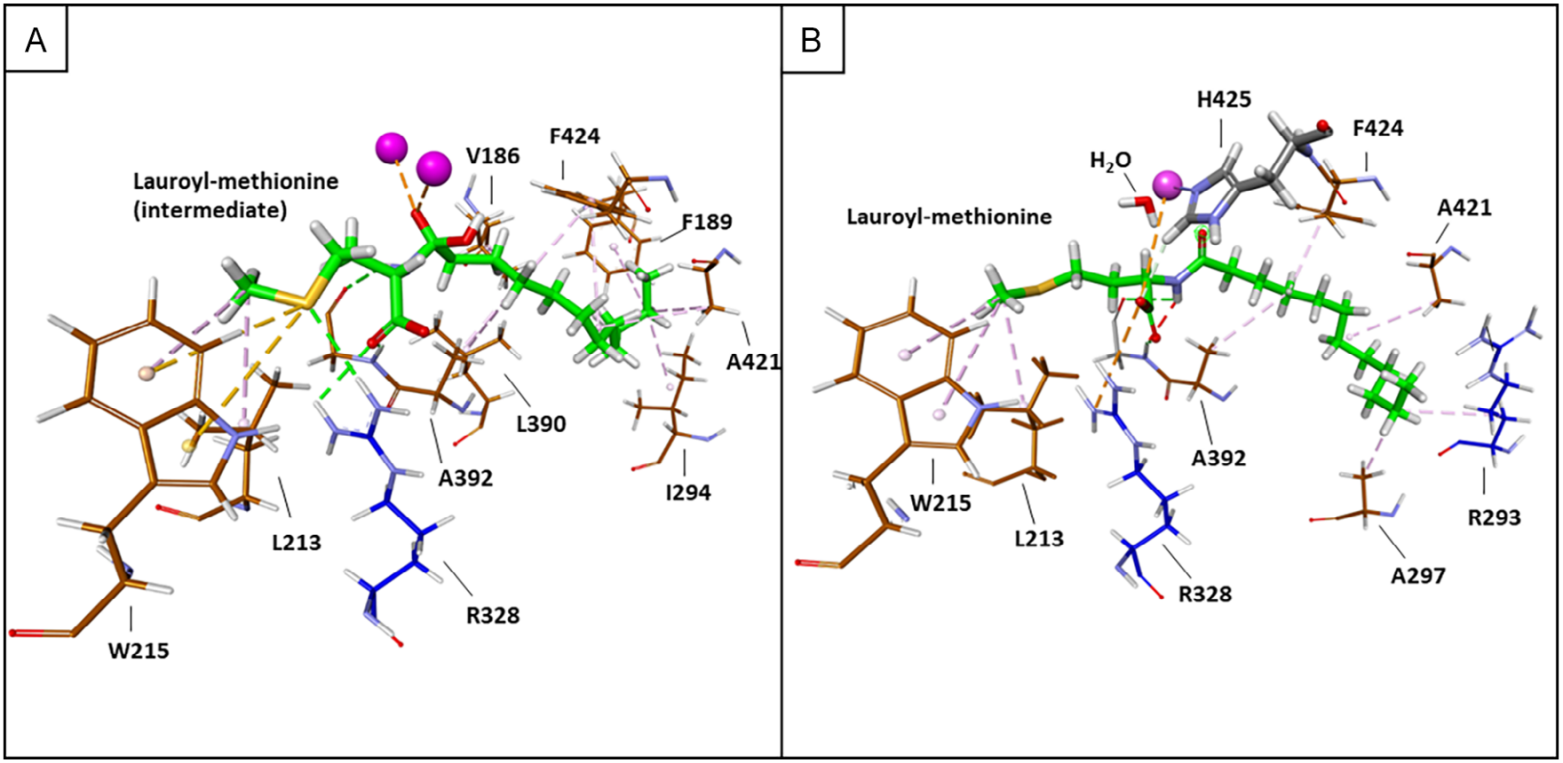
Structural visualization of docking the reaction intermediate of *N*‐lauroyl‐L‐methionine as well as the product to MsAA. The ligand interactions are shown as dashed lines (green for H‐bonds, orange for electrostatic interactions, lilac for hydrophobic interactions, and yellow for sulfur‐π bonds. (A) Docking of tetrahedral *N*‐lauroyl‐L‐methionine‐intermediate (green carbon atoms) to monomeric MsAA. (B) Docking of *N*‐lauroyl‐L‐methionine (green carbon atoms) to monomeric MsAA.

In the acylation reaction, after binding of lauric acid and methionine to the active site, the amino acids nitrogen attacks the carbonyl carbon of the fatty acid, so that a tetrahedral intermediate is formed. This intermediate breaks down to the acylated amino acid, in this case methionine, and water. In order to follow the condensation reaction in silico, and to get a better understanding of substrate binding during the reaction steps, a docking simulation was performed with the tetrahedral intermediate of lauroyl‐methionine. As shown in Figure 6A, the negative charge of the oxygen atom is stabilized by the zinc ions. The influence of H226(B)_MsAA_ could not be investigated in the model, as the docking of the intermediate was only successful with the monomeric structure. So, we tested the function of this residue by site‐directed‐mutagenesis (H226A). The residues binding the acyl‐ and amino acid parts of the tetrahedral intermediate are similar, compared to the previous docking with methionine and lauric acid. However, the position of methionyl has been slightly shifted towards the active site and lauric acid. This led to more in silico interactions between the substrate's thioether group and R328_MsAA_ (hydrogen bond) and W215_MsAA_ (sulfur‐π) (Table 2).

To perform the docking with lauroyl‐methionine, the second product of the condensation reaction, a water molecule was added to the protein structure. The position of the water molecule was taken from the crystal structure of HiDapE (PDB 3IC1) by first superimposing the two structures, copying the water molecule, which was located between the two zinc ions, and performing an energy minimization to position the molecule properly. Afterward, *N*‐lauroyl‐L‐methionine was docked to the enzyme with the bound water molecule. Again, as in the docking with lauric acid and methionine, or with the intermediate structure, the interacting protein side chains were the same (Figure 6B).

In summary, the protein side chains of MsAA interacting with the ligands in various stages of the condensation reaction were similar. The substrate pocket shows a distinct hydrophobic and hydrophilic side for the lauric acid and methionine moiety, respectively. The specificity for methionine, or bias towards small, hydrophobic amino acids, might be mediated by L213_MsAA_ and L357_MsAA_, and possibly W215_MsAA_. These residues are exchanged for S181_HiDapE_ and S290_HiDapE_ in HiDapE_HiDapE_, and W215_MsAA_ is exchanged for T183_HiDapE_, which does not interact with the ligand in the HiDapE structure PDB 5VO3. However, R328 is conserved in HiDapE and, like in the docking with MsAA, binds the carboxyl group adjacent to the nucleophilic amino group of the ligand. The results of the docking study, compared to the co‐crystallized structure of HiDapE with its bound substrates, revealed a very similar orientation of the substrates. The differences in substrate‐interacting residues that were observed and pointed out could explain the differences in the substrate scope.

### 2.9 Site‐Directed‐Mutagenesis of MsAA

**FIGURE 7:**
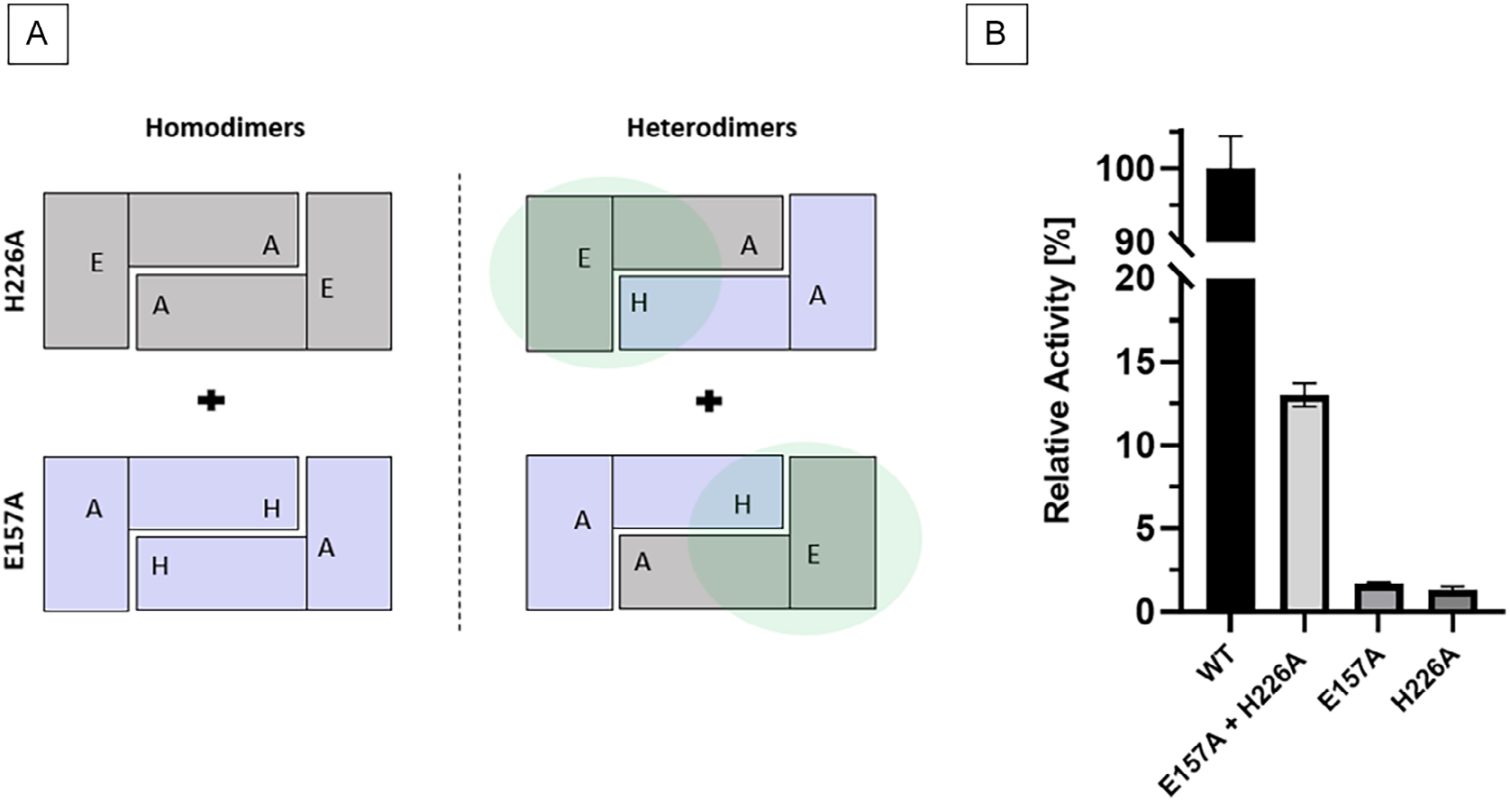
Enzyme complementation assay of variants E157A and H226A. (A) Schematic representation of the formation of homo‐ and heterodimers of MsAA E157A and H226A. Positions labeled ‘H’ and ‘E’ represent H226_MsAA_ and E157_MsAA,_ respectively. ‘A’ represents the mutation of these residues to alanine. The green circle represents an intact active site. (B) Relative hydrolytic activity of E157A and H226A as well as the combined variants to wild type MsAA toward *N*‐acetyl‐L‐alanine after 24 h incubation at 30°C. All samples were incubated at equal total protein concentration of 0.7 mg/ml.

The comparison of MsAA with structural homologs provided valuable insights into possibly functional residues. To confirm the catalytic roles of E157_MsAA_ and H226_MsAA_, site‐directed‐mutagenesis was performed. Following the same expression and purification protocol as for wild type MsAA the variants E157A and H226A were equally expressed and purified (Figure S12, S13). Albeit they only retained poor activities toward *N*‐acetyl‐L‐alanine hydrolysis of 1.7% (2.1 ± 0.1 U/mg) and 1.3% (1.6 ± 0.2 U/mg), respectively, compared to wild type MsAA (122 ± 5.2 U/mg). This strongly reduced activity indicates the role of E157_MsAA_ as a general acid/base. For both HiDapE and hAcy1, the glutamic acid residue is conserved as E147_hAcy1_ and E134_HiDapE_. Mutation of the general acid base catalyst to alanine led complete inactivity for HiDapE [22] and a 1000‐fold decrease in activity for hAcy1 [31]. In a Zn‐dependent thermolysin‐like protease from *Bacillus cereus* a glutamic acid which was thought to be an acid–base catalyst as well was mutated to serine. In this case also a residual activity (0.16%) was found and explained with the binding of a water molecule which takes over the catalytic function which might also be the reason for the residual activity of E157A_MsAA_ [32]. Lindner et al*.* demonstrated that H206_hAcy_, which is conserved as H226_MsAA_, acts in trans at the dimer interface and reaches into the active site of the other monomer. This was tested by performing an enzyme complementation assay and the formation of catalytically active heterodimers (Figure 7A) [26, 31]. To validate the role of H226_MsAA_ to act in trans of the other monomer, the variants E157A and H226A were incubated at equal protein amounts at 30°C for 24 h and the standard hydrolytic activity assay was performed. Evidently, heterodimers were formed since hydrolytic activity was restored to 15.9 (±0.5) U/mg, which is 13% of the wild‐type activity (Figure 7B). Mutations of the substrate‐binding residues L357_MsAA_ (L357S) and L213_MsAA_ (L213S + L357S) were performed as well, in accordance to the amino acid binding residues S181 and S290 of HiDapE (Table 2). They were equally expressed and purified as the wildtype (Figure S14, S15). However, none of the tested variants expanded the substrate scope or achieved higher conversions than the wild type in initial synthesis experiments (data not shown).

### 2.10 Proposed Mechanism for Synthesis of N‐Lauroyl‐L‐Methionine by MsAA

**FIGURE 8:**
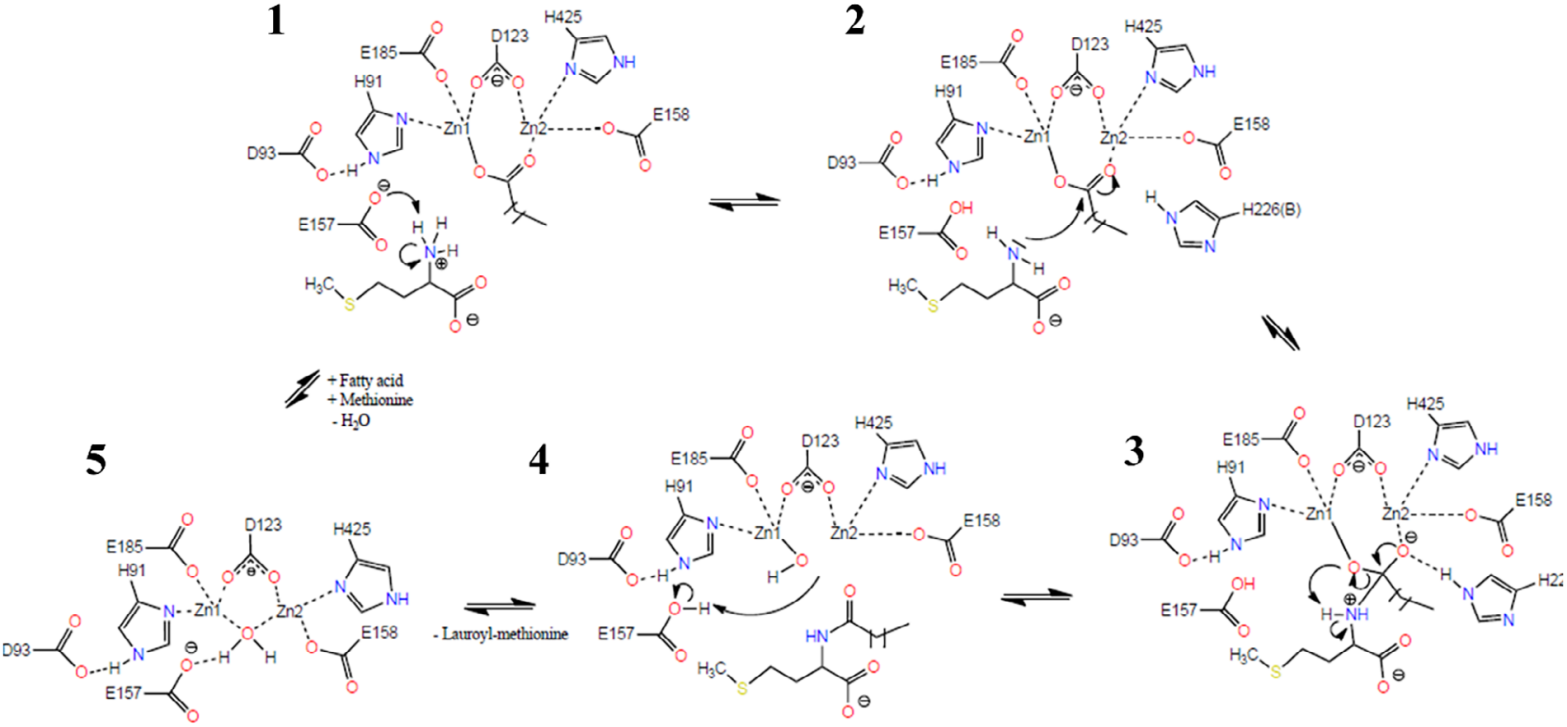
Proposed catalytic mechanism for the condensation of a fatty acid and methionine by aminoacylase MsAA. Step 1 shows binding of the methionine and lauric acid substrate to the active site, and the first reaction step of deprotonating the α‐amino group of methionine by E157. Step 2 comprises the nucleophilic attack under formation of the tetrahedral intermediate, stabilized by H226(B). In step 3, the complex decomposes under formation of a zinc‐bound hydroxide ion. The hydroxide ion is protonated by E157 in step 4. Step 5 shows the water molecule between the two zinc ions. When new methionine and lauric acid is added, the lauric acid replaces the water molecule in the active site and the reaction is reinitiated at step 1.

Based on the results obtained by docking, and by comparison with the proposed mechanism for hydrolysis of *N*‐succinyl‐L,L‐diaminopimelic acid by HiDapE [22], we proposed a mechanism for the synthesis of *N*‐lauroyl‐L‐methionine from methionine and lauric acid by MsAA (Figure 8).

The reaction may be initiated by lauric acid replacing the water molecule that is bound by Zn1 and Zn2. The negative charge of the deprotonated lauric acid is stabilized by the zinc ions, and, as it has been shown for HiDapE [22], the acidic oxygen of the substrate can be bound by Zn1, while the carbonyl oxygen is stabilized by Zn2. The catalytic E157_MsAA_ can deprotonate the amino group of the amino acid, so that it can perform a nucleophilic attack on the carbonyl carbon. This results in the formation of a tetrahedral complex and addition of a negative charge of the carbonyl oxygen. Again, as it has been described for HiDapE, H226(B)_MsAA_ could form an oxyanion hole to stabilize the intermediate. The complex decomposes under formation of *N*‐lauroyl‐L‐methionine and the zinc‐bound hydroxide, which can subsequently be protonated by E157 to form water.

## 3 Methods

### 3.1 Chemicals

The cultivation media, amino acids, metal salts, solvents, and tris(hydroxymethyl)aminomethane (Tris) were obtained from Carl Roth (Germany). *N‐*acetyl‐L‐alanine was purchased from Sigma–Aldrich (USA). The other acyl‐amino acids used as a substrate or standard for analytics, were synthesized as described previously [18]. D‐desthiobiotin was obtained from Merck (Germany). The Biochrom (UK) EZ Nin reagent was purchased from Laborservice Onken (Germany). Fatty acids and remaining chemicals were obtained from Sigma–Aldrich.

### 3.2 Production of Aminoacylase MsAA

The aminoacylase MsAA has been recombinantly produced and purified as described before [18]. *E. coli* ArcticExpress (DE3) (Agilent Technologies, USA) carrying the plasmid pET28a MsAA NTag was cultivated in Terrific Broth autoinduction medium (2% tryptone from casein, 2.4% yeast extract, 25 mM Na_2_HPO_4_, 25 mM KH_2_PO_4_, 50 mM NH_4_Cl, 2 mM MgSO_4_, 5 mM Na_2_SO_4_, 0.5% glycerol (v/v), 0.5% lactose, and 0.05% glucose) without antibiotics for expression. The expression temperature was 30°C for the first 6 h and was lowered to 12°C for the following 24 h of expression. The cells were harvested and disrupted by sonication. The buffer used for lysis and purification was 100 mM Tris‐HCl pH 7.0 with 150 mM NaCl and 1 mM ZnCl_2_. Additives for lysis were 0.3 mg/mL lysozyme and 0.1% Triton X‐100. Recombinant MsAA was purified by Strep‐Tag II affinity chromatography and elution from Strep‐Tactin SuperFlow high capacity cartridge (IBA, Germany) was done with 2.5 mM D‐desthiobiotin. The eluted protein solution was rebuffered without desthiobiotin using Vivaspin centrifugal filters (10,000 MWCO; Sartorius, Germany).

### 3.3 Mutagenesis of MsAA Aminoacylase

All active site variants of MsAA (E157A, H226A, L213S + L357S, and L357S) were cloned by site‐directed mutagenesis using primers that carried the specific mutation (Table S2) and enabled the insertion *via* Golden Gate cloning into the pET28‐eforRED backbone, as previously described [18].

### 3.4 Aminoacylase Activity Assay

Activity of aminoacylases was assayed by quantification of released amino acids with a ninhydrin‐based assay as previously described [33]. Briefly, 10 µL sample from amino acid solutions or aminoacylase reactions were mixed with 100 µL of EZ Nin:DMSO reagent, heated for 10 min at 99°C and diluted with 100 mM Na‐borate buffer pH 10.0 for measurement. In general, 200 µL reactions consisted of 190 µL substrate solution and 10 µL enzyme solution. For standard hydrolysis activity measurement, reaction with 15 mM *N*‐acetyl‐L‐alanine in 100 mM Tris‐HCl buffer pH 7.0 were performed at 30°C for 5 min. At 1 min sampling intervals, 10 µL samples were withdrawn and assayed with the ninhydrin assay. The absorbance of diketohydrindylidene‐diketohydrindamine, also called Ruhemann's purple, was determined at 570 nm with the Infinite M Nano absorbance plate reader (Tecan Group Ltd., Männedorf, CHE). One unit of MsAA was defined as the amount of enzyme that hydrolyzes one µmol of *N*‐acetyl‐L‐alanine per minute under the given conditions, respectively.

### 3.5 Biocatalytic Synthesis of Acyl‐Amino Acids

Initial biocatalytic synthesis of *N*‐lauroyl‐L‐methionine was investigated with 100 mM L‐methionine and 100 mM lauric acid in 100 mM Tris‐HCl pH 7.0 at 40°C for 72 h, without agitation. Generally, reactions were initiated by adding 0.02 mg/mL (1.3 U) of MsAA to a reaction volume of 0.5 mL to the reaction mixture. To stop the reaction, a 100 µL sample was withdrawn and immediately mixed with 100 µL of a mixture of 80% acetonitrile and 20% water containing 0.1% trifluoroacetic acid (TFA). The optimal pH for the synthesis reaction was determined from the reaction of 100 mM methionine and 100 mM lauric acid in 100 mM Tris‐HCl, adjusted to various pH values at 40°C, with analysis after 72 h. All further reactions were conducted in 100 mM Tris‐HCl at pH 8.0 without further adjustment during the reaction. The influence of reaction temperature on product formation was investigated in a range of 25°C to 60°C and was analyzed after 72 h. The substrate solution consisted of 100 mM methionine and 100 mM lauric acid.

The dependence of substrate concentrations for final product formation was investigated in a range of 25–400 mM for methionine and 25–200 mM for lauric acid. The respective other substrate was either kept constant at 100 mM or changed equimolarly, the latter only up to 200 mM concentration of lauric acid. The reactions were conducted at 45°C and analyzed after 24 h. In the case of 400 mM methionine, concentrations of either 100, 150, 200, or 300 mM lauric acid were used for synthesis, to determine the optimal ratio of the two substrates. For the acylation of alanine, isoleucine, leucine, phenylalanine and valine, the amino acid concentration was varied from 25–200 mM, while the concentration of lauric acid was kept at 100 mM. The reactions were conducted at 45°C for 24 h. The acyl‐donor specificity was investigated by employing 200 mM methionine with 100 mM of various fatty acids. As acyl donors, caprylic acid, decanoic acid, undecanoic acid, 10‐undecenoic acid, lauric acid, myristic acid, palmitic acid, and oleic acid were investigated. To increase solubility, 10% (v/v) EtOH was added to the substrate solutions. The reactions were conducted at 45°C for 24 h.

It was investigated if upscaling of the reaction had an influence on productivity. One reaction consisted of 1 mL and was conducted in 1.5 mL reaction tubes in a thermoshaker (Thermomix C; Eppendorf, Germany) at 40°C and 500 rpm. The upscaled reaction was conducted in a 100 mL glass spinner flask (Wheaton Celstir; DWK Life Sciences, Germany) at 40°C with magnetic stirring set to 500 rpm.

## 4 Analytical Methods

### 4.1 HPLC‐Ultraviolet Radiation (UV) and ‐ELSD Analysis

The quantitative analysis of the acyl‐amino acid synthesis reactions was performed by HPLC separation and UV and ELSD measurement. The HPLC system used was an Sykam S5200/S2100 (Germany) equipped with an ISAspher 100–5 C18 BDS column (C18, 5 µm, 4.0 * 250 mm; Isera, Germany) coupled with a Sykam UV Detector 2500 (Germany). An isocratic separation with 80% acetonitrile, 20% water and 0.1% TFA was performed at 1 mL/min flow rate and a column temperature of 40°C. The UV absorption of the eluent was measured at 210 nm. The product concentrations were calculated with an external standard of lauroyl‐amino acids, which have been chemically synthesized via Schotten–Baumann acylation as described previously [18]. The fatty acid spectrum was analyzed with a Shimadzu Nexera XR‐System equipped with a Hitachi LaChrom II‐column (C18, 5 µm, 4.6 x 250 mm) coupled with a VWR ELSD 100 detector. ELSD calibration was carried out using a log–log calibration model.

### 4.2 Mass Spectrometry Analysis

Qualitative and semiquantitative analysis of various amino acid acylation products was performed on an HPLC‐photodiode array (PDA)‐high‐resolution mass spectrometry (HRMS) system equipped with a PDA detector and an Orbitrap ID‐X Tribrid MS (Thermo Scientific). Separation was performed at a flow rate of 0.2 mL/min on an Alltima C18 column (2.1 × 100 mm, 3 μm, Hichrom) with methanol/water (80:20, v/v) supplemented with 0.1 % TFA as phase A and methanol supplemented with 0.1% TFA as solvent B, using a 10 min linear gradient elution from 0 to 98% solvent B. MS was performed in alternating positive/negative electrospray ionization mode (ESI^+/‐^) with the following conditions: spray voltage was set at 3.5 kV in ESI^+^ and −2.5 kV in ESI^‐^; source gases were set (in arbitrary units/min) for sheath gas, auxiliary gas and sweep gas at 35, 7, and 10, respectively; vaporizer temperature and ion transfer tube temperature were both set at 300°C. MS scans were performed from 100 to 500 m/z, at 7.5 K resolution (full width of the peak at its half maximum, fwhm, at 200 m/z) with parameters as follows: RF‐lens, 35%; maximum injection time, 50 ms; data type, profile; AGC target: 100 000; normalized AGC target: 25%. MS data acquisition and treatment were carried out utilizing the Xcalibur v. 3.0 software (Thermo Scientific).

### 4.3 Molecular Docking Simulations

The Discovery Studio 2021 suite from Daussalt Systemes Biovia (France) was used to perform molecular simulations. All simulations were conducted under the CHARMM force field [34]. The target molecule for the docking simulations was either the dimeric or monomeric MsAA structure, as predicted by the ColabFold (AlphaFold) algorithm [23]. As previously described [18], two zinc ions were added to the structure's active site with the Metal Ion‐Binding Site Prediction and Docking Server [35]. Lauric acid, L‐methionine, *N*‐lauroyl‐L‐methionine, and a tetrahedral intermediate of the latter molecule were non‐covalently docked against the targets. The docking simulations were performed with the CDOCKER method [36].

## Supporting Information

Additional supporting information can be found online in the Supporting Information section. **Supporting Fig. S1**: N‐lauroyl‐L‐alanine (molecular weight = 271.4 g/mol). MS1(A) and MS2 (B) spectra. **Supporting Fig. S2**: N‐lauroyl‐L‐valine (molecular weight = 299.4 g/mol). MS1(A) and MS2 (B) spectra. **Supporting Fig. S3**: N‐lauroyl‐L‐phenylalanine (molecular weight = 347.5 g/mol). MS1(A) and MS2 (B) spectra. **Supporting Fig. S4**: N‐lauroyl‐L‐leucine (molecular weight = 313.5 g/mol). MS1(A) and MS2 (B) spectra. **Supporting Fig. S5**: N‐lauroyl‐L‐isoleucine (molecular weight = 313.5 g/mol). MS1(A) and MS2 (B) spectra. **Supporting Fig. S6**: N‐capryloyl‐L‐methionine (N‐octanoyl‐L‐methionine; molecular weight = 275.4 g/mol). MS1(A) and MS2 (B) spectra. **Supporting Fig. S7**: N‐decanoyl‐L‐methionine (molecular weight = 303.5 g/mol). MS1(A) and MS2 (B) spectra. **Supporting Fig. 8**: N‐10‐undecenoyl‐L‐methionine (molecular weight = 315.5 g/mol). MS1(A) and MS2 (B) spectra. **Supporting Fig. S9**: N‐lauroyl‐L‐methionine (molecular weight = 331.5 g/mol). MS1(A) and MS2 (B) spectra. **Supporting Fig. S10**: N‐palmitoyl‐L‐methionine (molecular weight = 387.6 g/mol). MS1(A) and MS2 (B) spectra. **Supporting Fig.S 11**: N‐oleoyl‐L‐methionine (molecular weight = 413.7 g/mol). MS1(A) and MS2 (B) spectra. **Supporting Fig. S12**: SDS‐PAGE analysis of MsAA E157A overexpression and purification via Strep‐tag from E. coli BL21(DE3) with autoinduction at 20°C and incubation for 24 h at 220 rpm. Lane 1: Protein marker (BlueEasy Prestained Protein Marker, Nippon Genetics); lane 2: cell‐free extract; lane 3: Flow‐Through; lane 4: insoluble fraction; lane 5: first wash fraction; lane 6: last wash fraction; lane 7: elution of MsAA E157A; lane 8: protein marker. **Supporting Fig. S13**: SDS‐PAGE analysis of MsAA H226A overexpression and purification via Strep‐tag from E. coli BL21(DE3) with autoinduction at 20°C and incubation for 24 h at 220 rpm. Lane 1: Protein marker (BlueEasy Prestained Protein Marker, Nippon Genetics); lane 2: cell‐free extract; lane 3: insoluble fraction; lane 4: flow‐through; lane 5: wash fraction; lane 6: elution of MsAA H226A. **Supporting Fig. S14**: SDS‐PAGE analysis of MsAA L213S overexpression and purification via Strep‐tag from E. coli BL21(DE3) with autoinduction at 20°C and incubation for 24 h at 220 rpm. Lane 1: Protein marker (BlueEasy Prestained Protein Marker, Nippon Genetics); lane 2: cell‐free extract; lane 3: flow‐through; lane 4: insoluble fraction; lane 5: wash first; lane 6: wash last; lane 7: elution of MsAA H226A. **Supporting Fig. S15**: SDS‐PAGE analysis of MsAA L213S + L357S overexpression and purification via Strep‐tag from E. coli BL21(DE3) with autoinduction at 20°C and incubation for 24 h at 220 rpm. Lane 1: Protein marker (BlueEasy Prestained Protein Marker, Nippon Genetics); lane 2: cell‐free extract; lane 3: insoluble fraction; lane 4: wash first; lane 5: wash last; lane 6: elution of MsAA H226A; lane 7: Protein marker. **Supporting Table S1**: Experimental data for the influence of glycerol on enzyme stability and substrate conversion. Reactions were performed with 400 mM methionine, 150 mM lauric acid, 100 mM Tris‐HCl, 50 µM ZnCl_2_, pH 8.0 at 40°C‐60°C for 72 h. All measurements were done in triplicates. **Supporting Table S2**: Primers used for site‐directed mutagenesis of MsAA.

## Funding

This study was supported by Bundesministerium für Bildung und Forschung (13FH256PB6).

## Conflict of Interest

The authors declare no conflicts of interest.

## Supporting information

Supplementary Material

## Data Availability

The data that support the findings of this study are available from the corresponding author upon reasonable request.
