# Supplementary material for: Biocatalytic Potential of a Mycobacterial Aminoacylase for Synthesis of N‐Acyl‐L‐Amino Acids in Aqueous Media
Source: Chembiochem. 2026 Apr 8;27(7):e70313. doi: 10.1002/cbic.70313 (PMC13058870; doi:10.1002/cbic.70313)
Supplement: Supplementary file 1 — Supplementary Material [file CBIC-27-e70313-s001.pdf]

Supplementary materials

Journal name:

ChemBioChem

Title:

Biocatalytic Potential of a Mycobacterial Aminoacylase for Synthesis of *N*-acyl-L-Amino Acids in Aqueous Media

Author's names:

Jessika Wirges[a], Gerrit Haeger[b], Laureline Gennesseaux[c], Yann Guiavarc'h[c], Catherine Humeau[c], Cédric Paris[d], Isabelle Chevalot[c], Karl-Erich Jaeger[e], Jonas Krapohl[a], Patrick Schmidt[a], Johannes Bongaerts[a], Petra Siegert[a]

Addresses:

[a]Institute of Nano- and Biotechnologies, Aachen University of Applied Sciences, 52428 Jülich, Germany
[b]Novonesis A/S, Production Strain Technology, Brudelysvej 26, 2880 Bagsværd, Denmark
[c]Université de Lorraine, CNRS, Laboratoire Réactions et Génie des Procédés (LRGP), F-54000, Nancy, France
[d]Université de Lorraine, CNRS, Laboratoire d'Ingénierie des Biomolécules (LIBio), F-54000, Nancy, France
[e]Institute of Molecular Enzyme Technology, Heinrich Heine University Düsseldorf, 52425 Jülich, Germany

Author for correspondence:

Petra Siegert: Heinrich-Mussmann-Str. 1, 52428 Jülich, Germany. E-mail address: siegert@fh-aachen.de (P. Siegert), Tel.: +49 241 6009 53124

LC-MS spectra of synthesized lauroyl-amino acids:

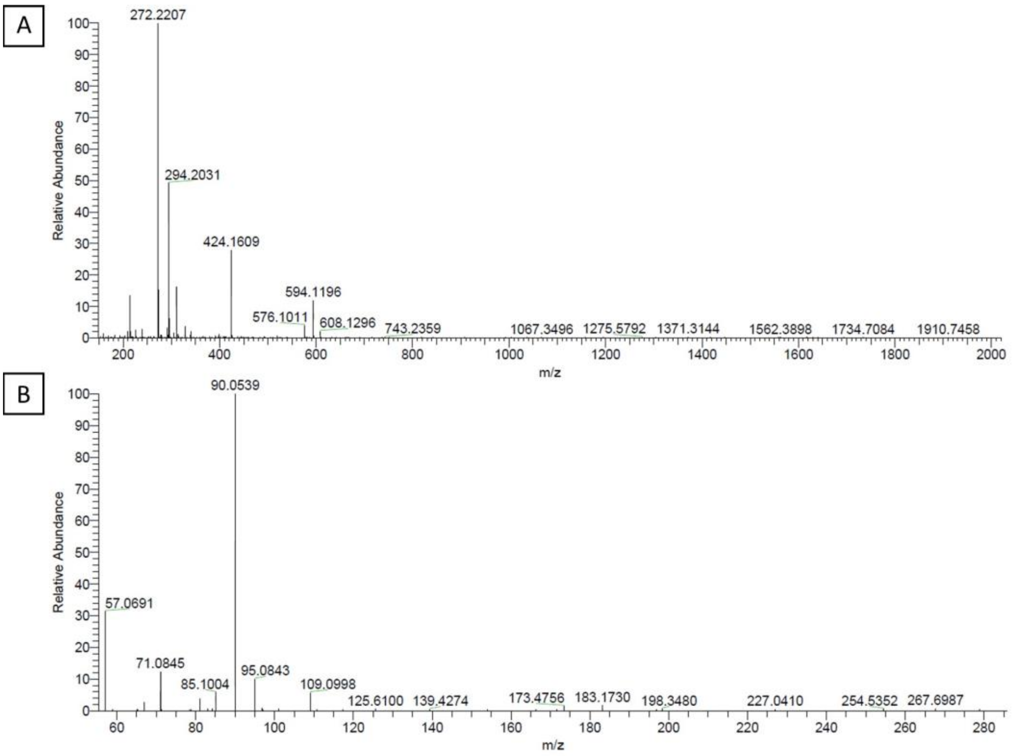

Figure S1: N-lauroyl-L-alanine (molecular weight = 271.4 g/mol). MS1(A) and MS2 (B) spectra.

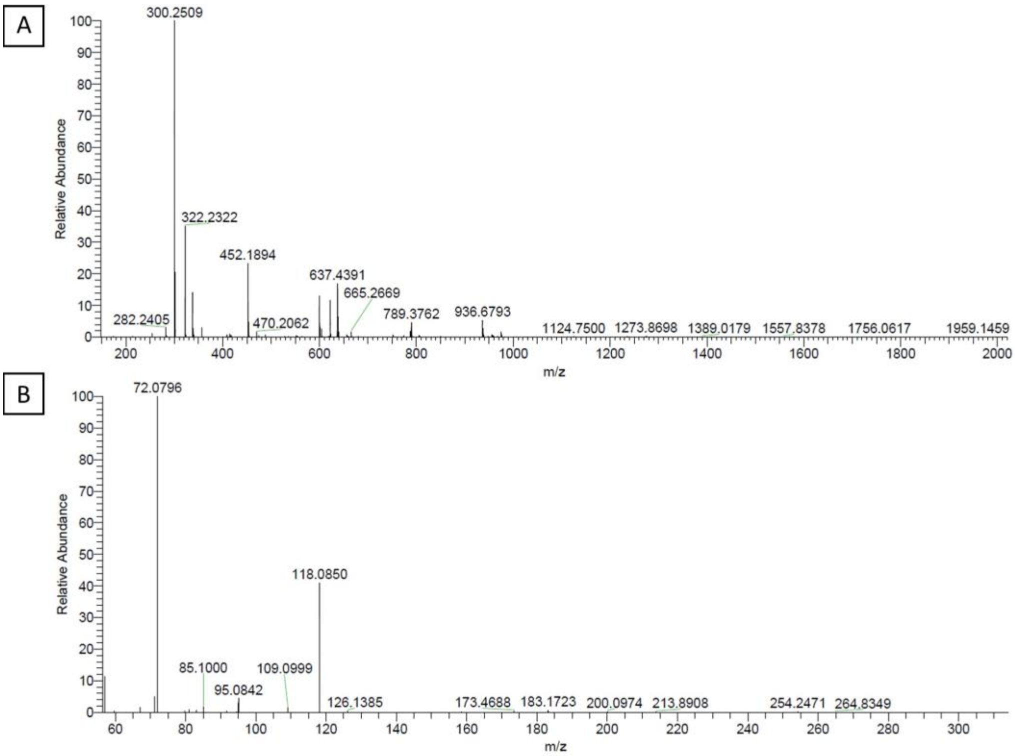

Figure S2: N-lauroyl-L-valine (molecular weight = 299.4 g/mol). MS1(A) and MS2 (B) spectra.

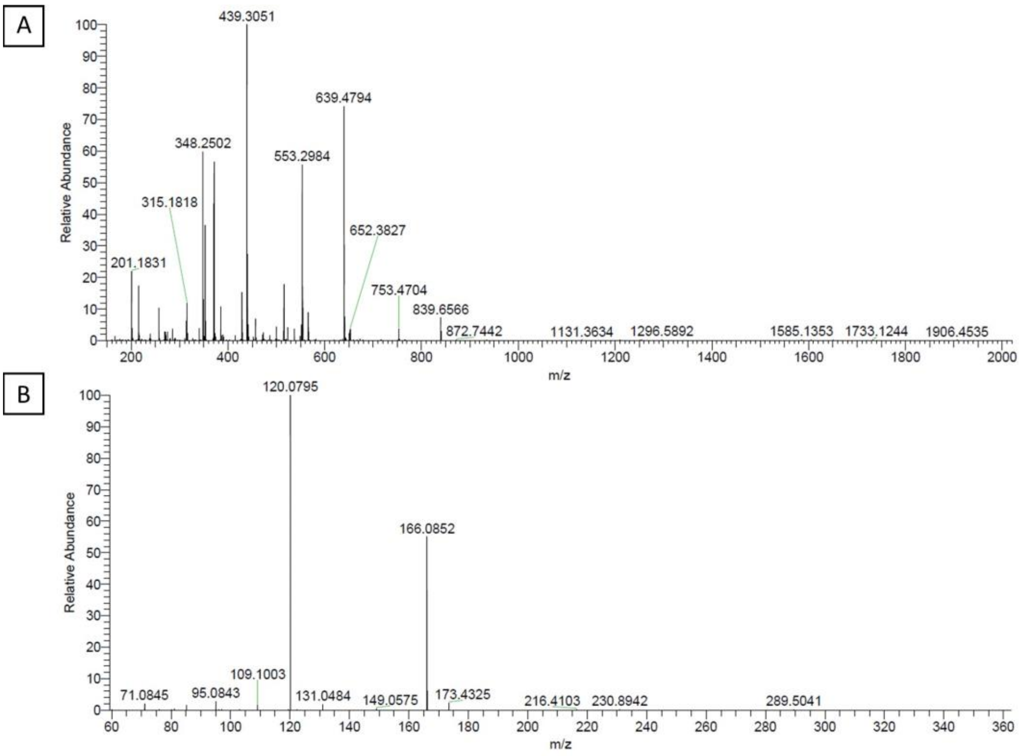

Figure S3: N-lauroyl-L-phenylalanine (molecular weight = 347.5 g/mol). MS1(A) and MS2 (B) spectra.

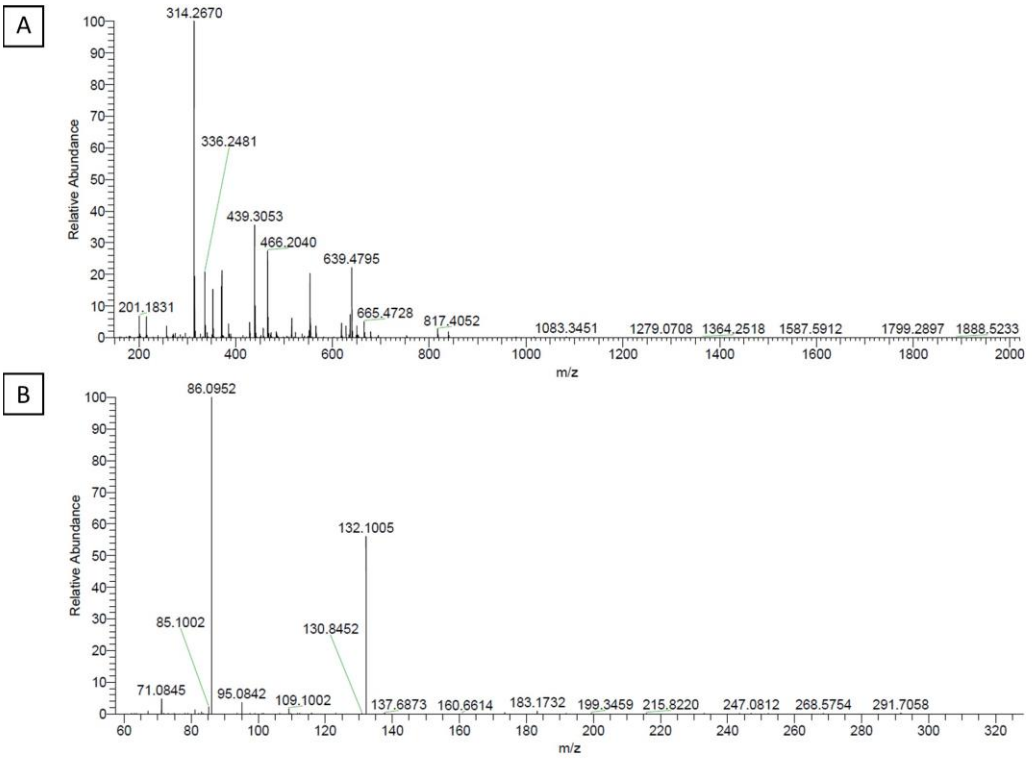

Figure S4: N-lauroyl-L-leucine (molecular weight = 313.5 g/mol). MS1(A) and MS2 (B) spectra.

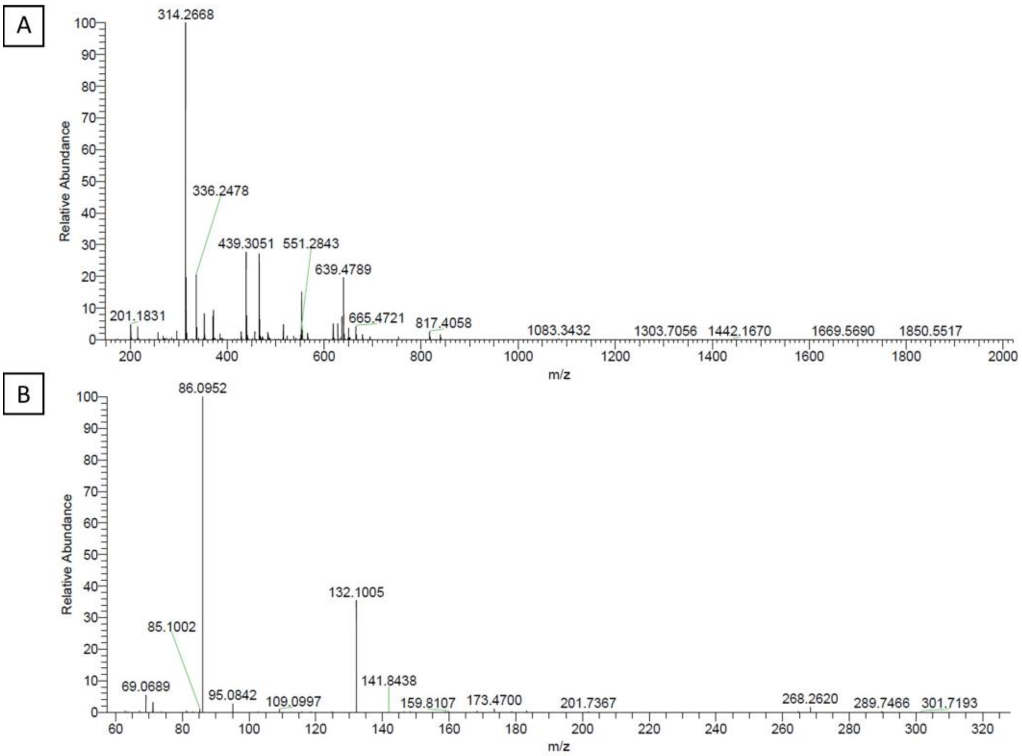

Figure S5: N-lauroyl-L-isoleucine (molecular weight = 313.5 g/mol). MS1(A) and MS2 (B) spectra.

LC-MS spectra of synthesized acyl-methionines:

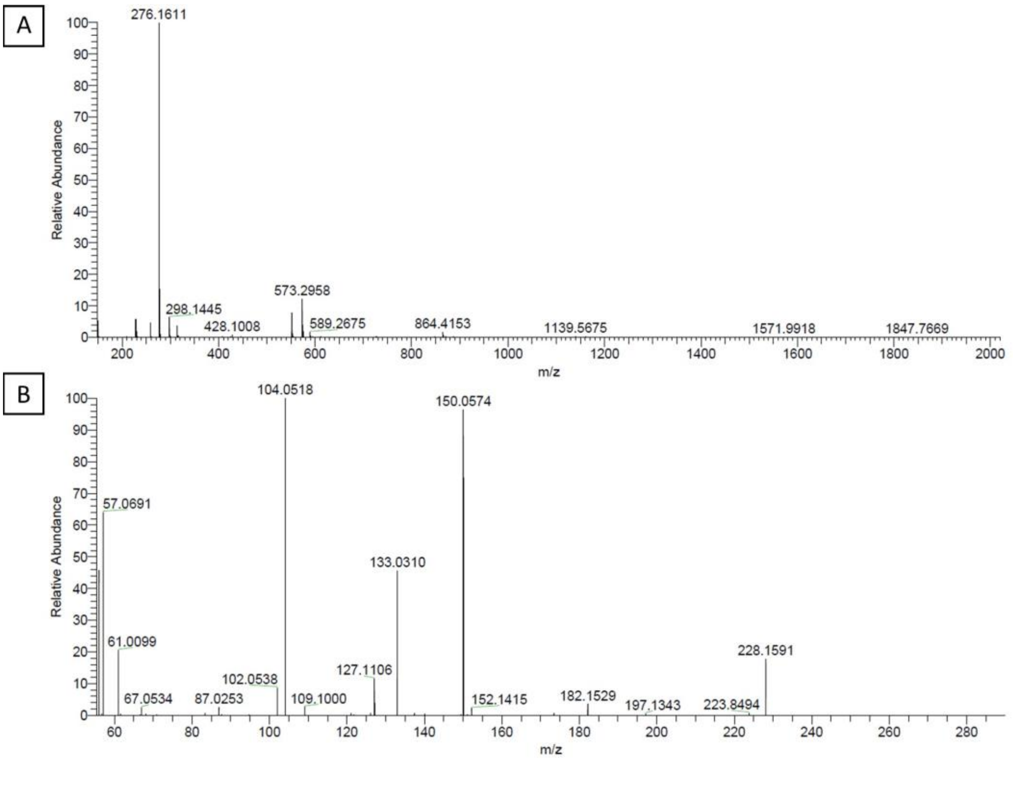

Figure S6: N-capryloyl-L-methionine (N-octanoyl-L-methionine; molecular weight = 275.4 g/mol). MS1(A) and MS2 (B) spectra.

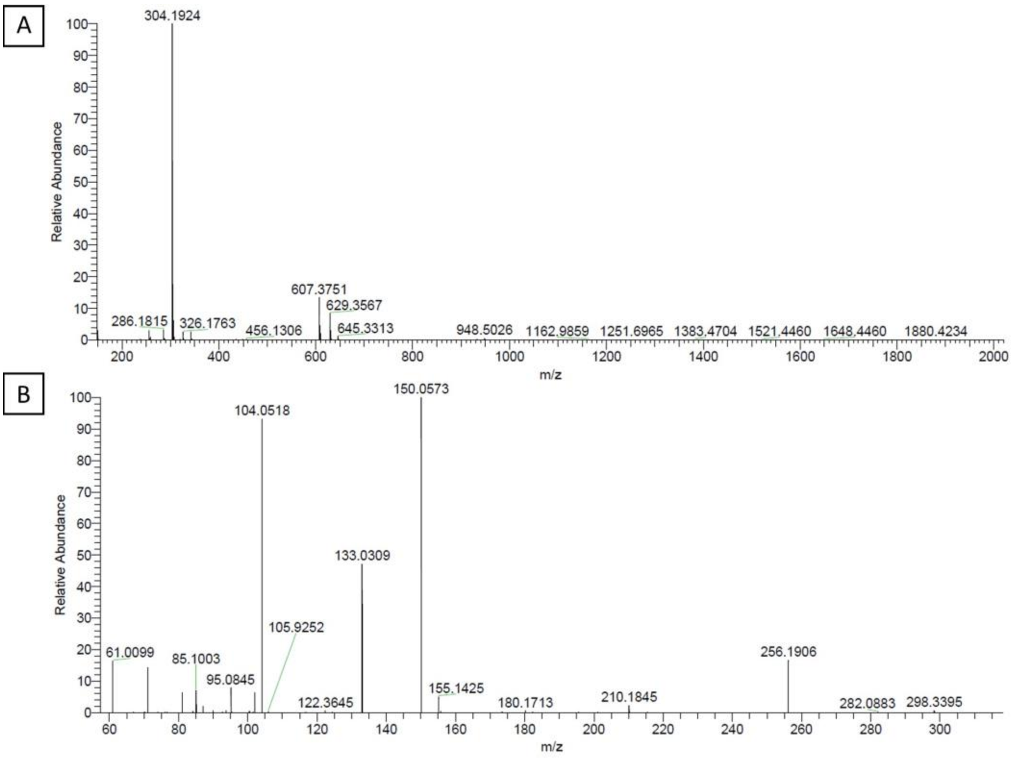

Figure S7: N-decanoyl-L-methionine (molecular weight = 303.5 g/mol). MS1(A) and MS2 (B) spectra.

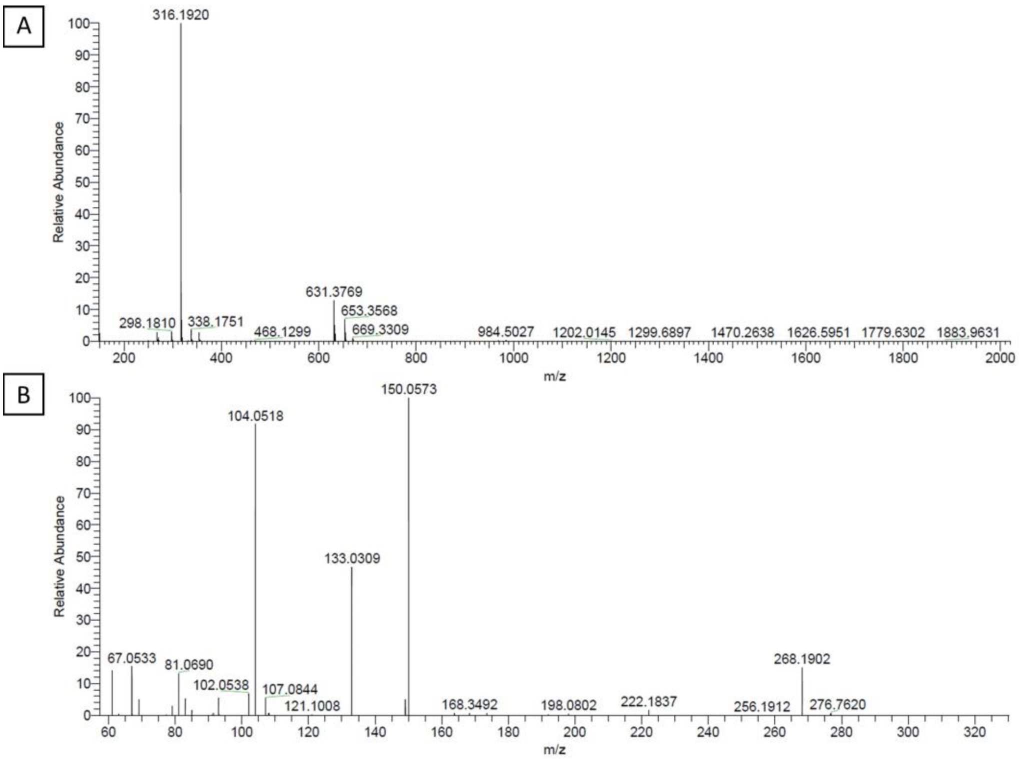

Figure S8: N-10-undecenoyl-L-methionine (molecular weight = 315.5 g/mol). MS1(A) and MS2 (B) spectra.

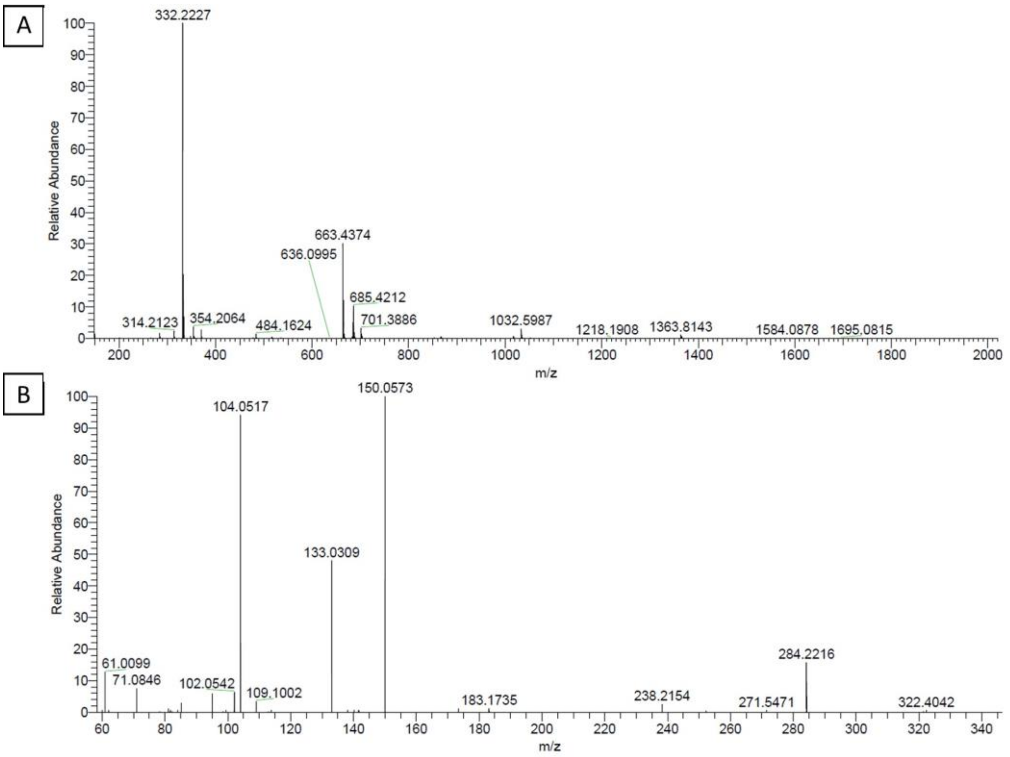

Figure S9: N-lauroyl-L-methionine (molecular weight = 331.5 g/mol). MS1(A) and MS2 (B) spectra.

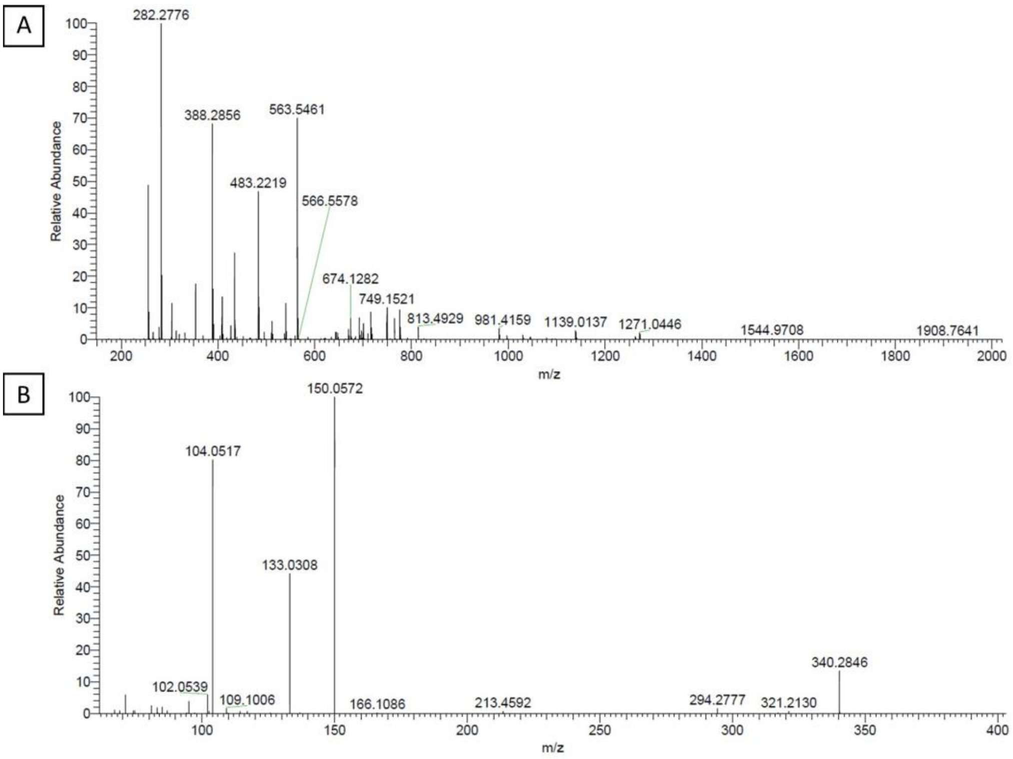

Figure S10: N-palmitoyl-L-methionine (molecular weight = 387.6 g/mol). MS1(A) and MS2 (B) spectra.

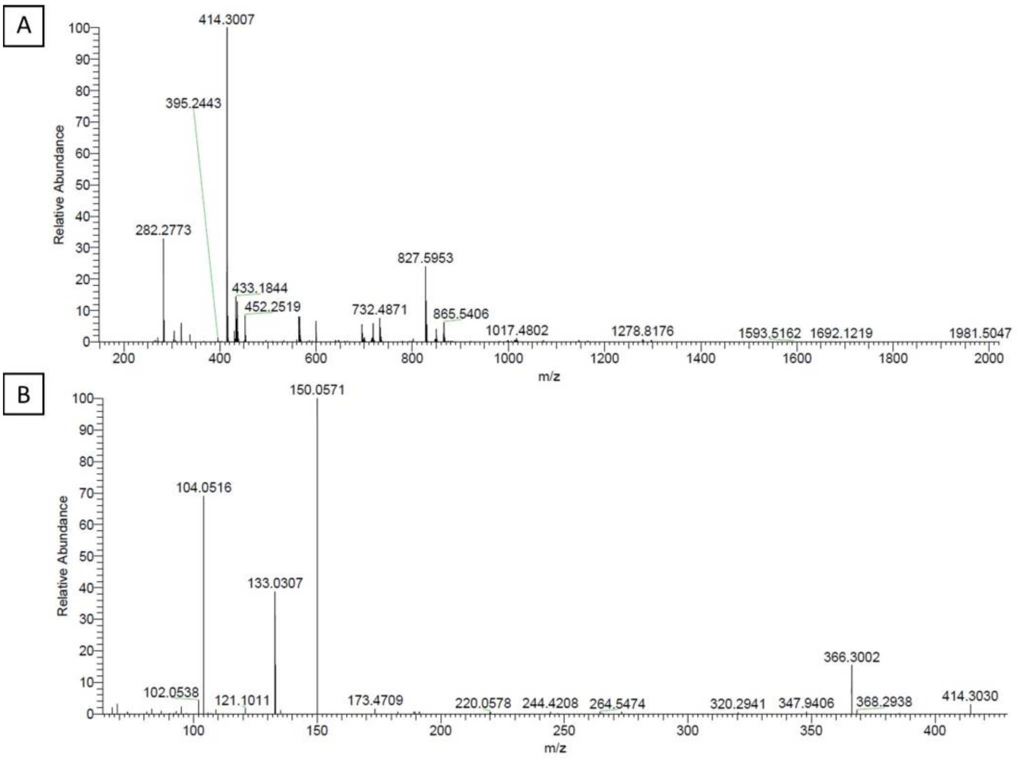

Figure S11: N-oleoyl-L-methionine (molecular weight = 413.7 g/mol). MS1(A) and MS2 (B) spectra.

Table S1: Experimental data for the influence of glycerol on enzyme stability and substrate conversion. Reactions were performed with 400 mM methionine, 150 mM lauric acid, 100 mM Tris-HCl, 50 µM $ZnCl_2$, pH 8.0 at 40-60°C for 72 h. All measurements were done in triplicates.

Temperature [°C]	Glycerin (w/v) [%]	*N*-lauroyl-methionine [mM]
40	0	65.1
40	15	75.1
40	30	76.0
50	0	24.6
50	15	75.1
50	30	78.3
60	0	2.4
60	15	14.4
60	30	37.1

Table S2: Primers used for site-directed mutagenesis of MsAA.

	Nucleotide sequence (5’ → 3’)
MsAA BsaI fw	GGTCTCCCATGTGGTCACATCCGCAGTTTGAAAAAAG
MsAA BsaI rev	GGTCTCTCTCAACAATTCTGCAGGAAATGTTCCAGAAC
MsAA E157A fragment 1 rev	GCCACCATGTTCCGCATCGGCAACAAATGC
MsAA E157A fragment 2 fw	GCCGATGCGGAACATGGTGGCACCTATGGTG
MsAA H226A fragment 1 rev	GCACCATGCTACCCGCACCTGCACGACCACGTG
MsAA H226A fragment 2 fw	GTCGTGCAGGTGCGGGTAGCATGGTGCATGATG

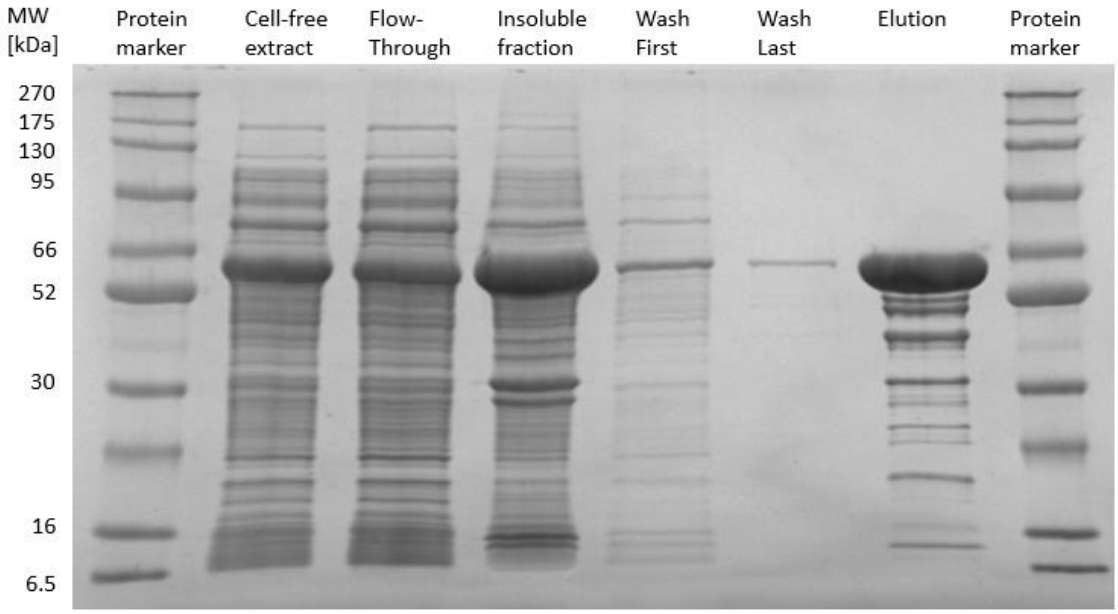

Figure S12: SDS-PAGE analysis of MsAA E157A overexpression and purification via Strep-tag from E. coli BL21(DE3) with autoinduction at 20 °C and incubation for 24 h at 220 rpm. Lane 1: Protein marker (BlueEasy Prestained Protein Marker, Nippon Genetics); lane 2: cell-free extract; lane 3: Flow-Through; lane 4: insoluble fraction; lane 5: first wash fraction; lane 6: last wash fraction; lane 7: elution of MsAA E157A; lane 8: protein marker.

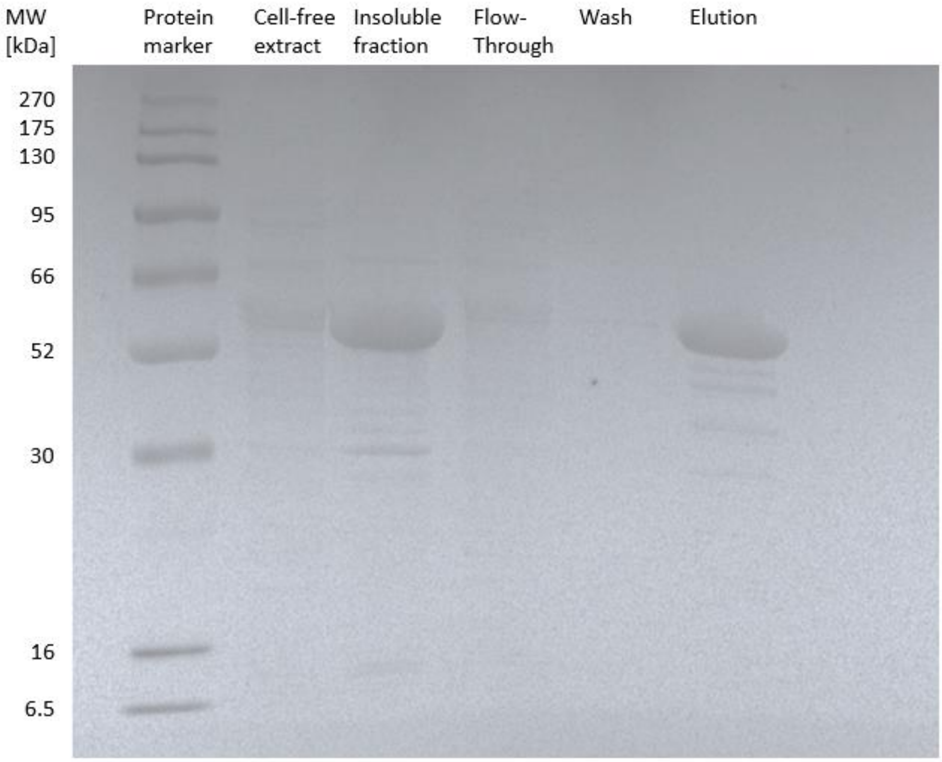

Figure S13: SDS-PAGE analysis of MsAA H226A overexpression and purification via Strep-tag from E. coli BL21(DE3) with autoinduction at 20 °C and incubation for 24 h at 220 rpm. Lane 1: Protein marker (BlueEasy Prestained Protein Marker, Nippon Genetics); lane 2: cell-free extract; lane 3: insoluble fraction; lane 4: flow-through; lane 5: wash fraction; lane 6: elution of MsAA H226A.

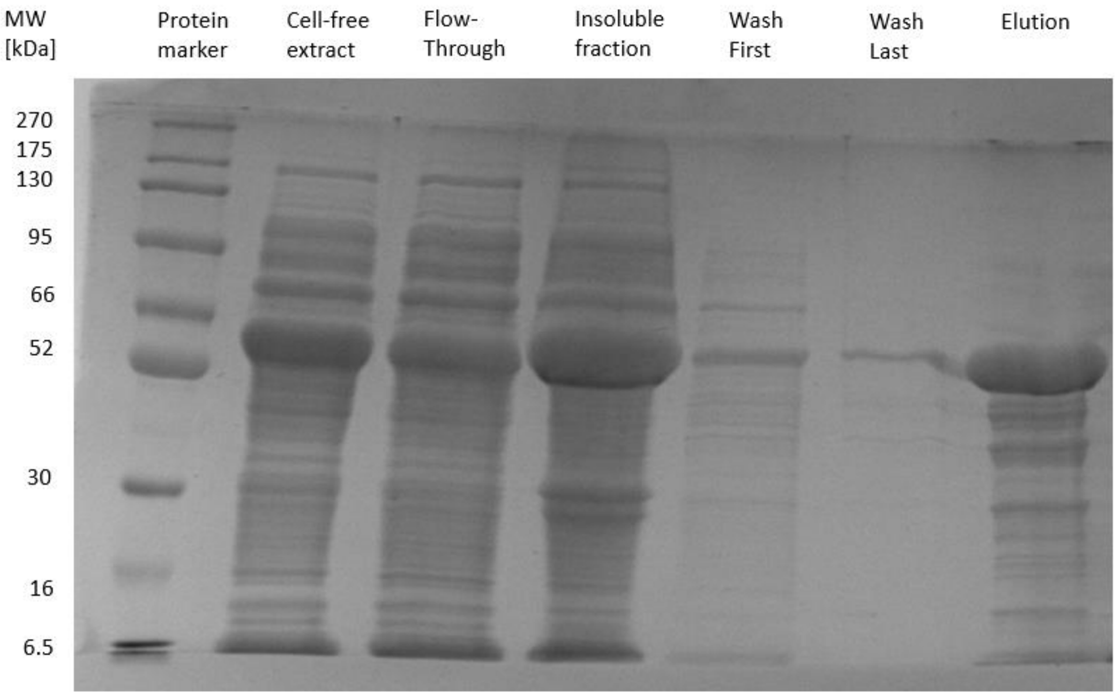

Figure S14: SDS-PAGE analysis of MsAA L213S overexpression and purification via Strep-tag from E. coli BL21(DE3) with autoinduction at 20 °C and incubation for 24 h at 220 rpm. Lane 1: Protein marker (BlueEasy Prestained Protein Marker, Nippon Genetics); lane 2: cell-free extract; lane 3: flow-through; lane 4: insoluble fraction; lane 5: wash first; lane 6: wash last; lane 7: elution of MsAA H226A.

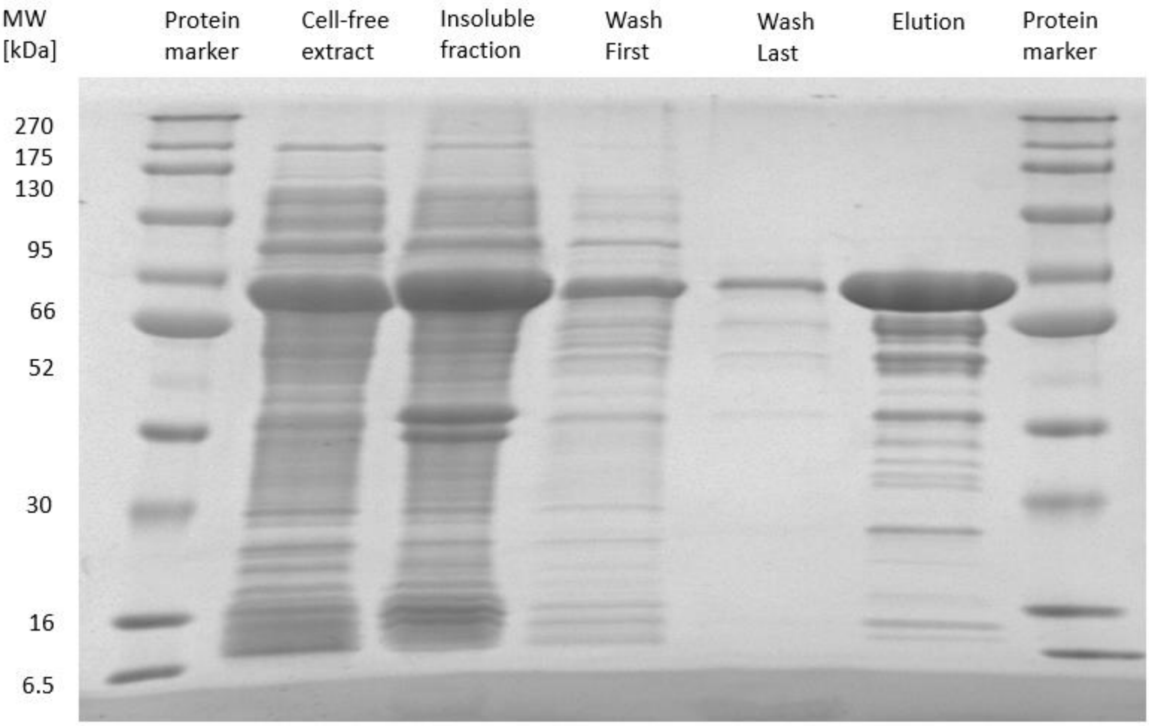

Figure S15: SDS-PAGE analysis of MsAA L213S + L357S overexpression and purification via Strep-tag from E. coli BL21(DE3) with autoinduction at 20 °C and incubation for 24 h at 220 rpm. Lane 1: Protein marker (BlueEasy Prestained Protein Marker, Nippon Genetics); lane 2: cell-free extract; lane 3: insoluble fraction; lane 4: wash first; lane 5: wash last; lane 6: elution of MsAA H226A; lane 7: Protein marker.
